# Single-digit-micrometer-resolution continuous liquid interface production

**DOI:** 10.1126/sciadv.abq2846

**Published:** 2022-11-16

**Authors:** Kaiwen Hsiao, Brian J. Lee, Tim Samuelsen, Gabriel Lipkowitz, Jason M. Kronenfeld, Dan Ilyn, Audrey Shih, Maria T. Dulay, Lee Tate, Eric S. G. Shaqfeh, Joseph M. DeSimone

**Affiliations:** ^1^ Department of Radiology, Stanford University, Stanford, CA 94305, USA.; ^2^ Department of Mechanical Engineering, Sungkyunkwan University, Suwon, Republic of Korea.; ^3^ Department of Mechanical Engineering, Stanford University, Stanford, CA 94305, USA.; ^4^ Department of Chemistry, Stanford University, Stanford, CA 94305, USA.; ^5^ Department of Chemical Engineering, Stanford University, Stanford, CA 94305, USA.; ^6^ Digital Light Innovations, Austin, TX 78728, USA.

## Abstract

To date, a compromise between resolution and print speed has rendered most high-resolution additive manufacturing technologies unscalable with limited applications. By combining a reduction lens optics system for single-digit-micrometer resolution, an in-line camera system for contrast-based sharpness optimization, and continuous liquid interface production (CLIP) technology for high scalability, we introduce a single-digit-micrometer-resolution CLIP-based 3D printer that can create millimeter-scale 3D prints with single-digit-micrometer-resolution features in just a few minutes. A simulation model is developed in parallel to probe the fundamental governing principles in optics, chemical kinetics, and mass transport in the 3D printing process. A print strategy with tunable parameters informed by the simulation model is adopted to achieve both the optimal resolution and the maximum print speed. Together, the high-resolution 3D CLIP printer has opened the door to various applications including, but not limited to, biomedical, MEMS, and microelectronics.

## Teaser

A high-resolution CLIP 3D printer can rapidly and scalably fabricate 3D structures with single-digit-micrometer resolution.

## INTRODUCTION

Unrestricted freeform manufacturing of three-dimensional (3D) structures on the mesoscale spanning the dimensions from single-digit micrometer to 1 cm (four orders of magnitude) has been a long-sought engineering challenge (*1*). The ability to form 3D structures with feature sizes in the microscopic length scale and hierarchical complexity in the macroscopic length scale offers powerful engineering design options for microsystem technologies where conventional planar architectures currently dominate (*2*, *3*). The paradigm of building microstructures via additive manufacturing (AM) is revolutionizing 3D fabrication, with relevance in biomedical devices (*4*–*6*), microelectromechanical systems (*7*, *8*), energy storage platforms (*9*), optoelectronic components (*10*, *11*), and microelectronics (*12*, *13*). This endeavor has pushed and explored the limits of previously unexplored 3D fabrication methods, with an aim of increasing throughput and resolution. Recent breakthroughs have also started to challenge well-established benchmarks in the microelectronics industry, including resolution, precision, feature size, and efficiency, which have been developed over more than 50 years (*14*). Moreover, AM brings the fundamental advantages of arbitrary designs in all Cartesian coordinates, whereas lithographic approaches are limited to X-*Y* planar and vertical patterned structures in the *z* direction, yielding products with only rectilinear features (e.g., microfluidics with channels having only rectangular cross sections and interlayer vias in microelectronics that are always vertical only as opposed to being on a diagonal).

AM fabrication approaches offer unprecedented customization and flexibility, with the capability to create complex structures that cannot be produced by traditional methods. However, AM also has its own new set of fabrication constraints. The three most prominent and common issues that render most AM technologies unscalable and difficult to compete with traditional lithography approaches are the layer artifacts that lead to mechanical anisotropy, reduced resolution, and slow build rates. The single-digit-micrometer-resolution continuous liquid interface production (CLIP)–based 3D printer reported here overcomes all three constraints simultaneously.

All AM technologies other than projection stereolithography rely on a serial printing process that results in slow build rate that often scales inversely to the print resolution, including two-photon polymerization (TPP) (*15*–*21*), inkjet printing (IJ) (*22*), fused deposition modeling (*23*, *24*), direct ink write (DIW) (*25*–*28*), selective laser sintering (SLS) and selective laser melting (SLM) (*29*, *30*). For example, the TPP technique has been powerful in driving the fabrication resolution down to the submicrometer length scale. However, it takes several weeks to create a centimeter-length-scale microarchitecture with TPP because of its slower print speed. Microstereolithography (PμSL) has substantially improved the projection resolution (*31*–*40*), and the recent large-area-projection PμSL technologies (*41*) have further increased the print speed and build area. The repeated step-expose-delamination process in projection stereolithography oftentimes results in layer artifacts such as degradation in surface characteristics, anisotropic properties of finished parts, slowing down of the printing process, and limitations on printing materials.

The leap in the continuous fabrication method introduced by 3D CLIP technology removes the layer-by-layer printing process through the introduction of an oxygen-permeable window that takes advantage of oxygen inhibition at the dead-zone (*42*). This technological breakthrough completely removes the delamination step in the printing process and has allowed it to noticeably increase the print speed by 100 times when compared to conventional projection-based stereolithography printers and 10^5^ times when compared to serial printing 3D printers. Specifically, the interlayer time between each layer for PμSL involves a delamination step and is typically in the range of 4 to 5 s (*43*), whereas for CLIP, the time ranges between 50 and 150 ms, leading to an increase in print speed by at least 100.

In the present manuscript, we describe a high-resolution CLIP technology that allows the fabrication of 3D structures containing single-digit-micrometer features at a print speed that is 10^5^ faster than commercially available high-resolution 3D printers (Nanoscribe). This is accomplished by combining the CLIP technology with a custom-designed projection optical lens and an in-line contrast-based focusing system. To maneuver the shallow depth of focus for a high-magnification objective lens, we developed a robust calibration platform to locate the optimal focal plane, thus resolving the fine details of the projected patterns with reproducibility. To achieve an understanding of photopolymerization kinetics on print resolution and the impact of resin transport on print speed for our system, we have introduced a numerical model that considers all fundamental elements in our high-resolution 3D CLIP printing system, including optical projection, photopolymerization reaction kinetics, and resin mass transport. This model allows us to develop a printing strategy that uses the understanding of fundamental transport phenomena and determine print parameters for the printer software control system. Aside from optimizing the print process, the model also provides fundamental insights into 3D CLIP printing in general, with accurate predictions of the surface finish of a printed part, dead-zone thickness, and resin curing during the 3D printing process. Together, we introduce a new single-digit-micrometer-resolution 3D CLIP-based printer with a custom-designed projection optics lens system, in-line focusing system, and a software-controlled printing process informed by parameters from our first principles–based model.

## RESULTS

### Projection optics system for achieving single-digit-micrometer-resolution

**Fig. 1.**
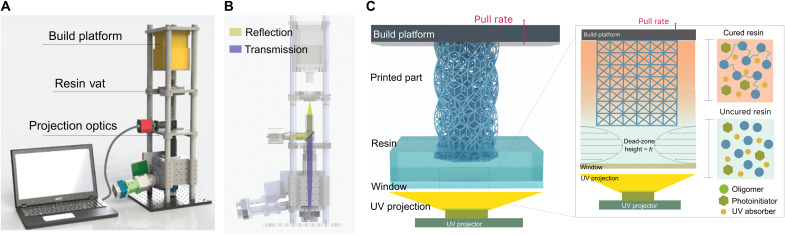
Single-digit-micrometer-resolution CLIP-based 3D printer setup schematic and printing process. (**A**) Schematic of the single-digit-micrometer-resolution CLIP-based 3D printer. The 3D printer consists of a UV projector, a projection lens, a resin vat that contains an oxygen-permeable window, and a translation stage. (**B**) Projection optics system includes a UV camera and a computer for real-time monitoring, where the projected UV light path (purple) is reflected through the beam splitter, and the reflected projection (yellow) is captured by the UV camera, thereby allowing for real-time monitoring of the projected images and enabling fine adjustment of the focal plane. (**C**) CLIP process contains an oxygen-permeable window, which is not only highly transmissive to UV (385 nm) but also permeable to oxygen. The permeated oxygen forms a thin layer of dead-zone above the window, where photopolymerization is inhibited, allowing a continuous 3D print.

The single-digit-micrometer-resolution CLIP-based 3D printer system has been designed and implemented in our laboratory. The system is based on a combination of the CLIP printing technology (*42*) and a reduction optics system to achieve fast print speed, smooth surface, and high-resolution print (Fig. 1A). The projection optics system consists of a tube lens and microscope objective with built-in magnifications of ×2 and ×5 to shrink the 7.6-μm native pixel size of the digital micromirror device (DMD) to 3.8 or 1.5 μm, respectively. A real-time projection pattern monitoring and focal plane adjustment system that contains a beam splitter and a charge-coupled device (CCD) camera is designed in the projection light path (Fig. 1B; details can be found in Materials and Methods).

The CLIP printing process is achieved through an oxygen-permeable window that creates a thin dead-zone that inhibits photopolymerization for continuous fabrication of printed parts (Fig. 1C). This dead-zone allows a continuous flow of resin replenishment, thus eliminating the delamination of the printed part from the window, which is known to be the rate-limiting step in stereolithography (SLA), digital-light-processing (DLP), and PμSL.

### Contrast-based focusing mechanism to optimize the focal plane and print resolution

**Fig. 2.**
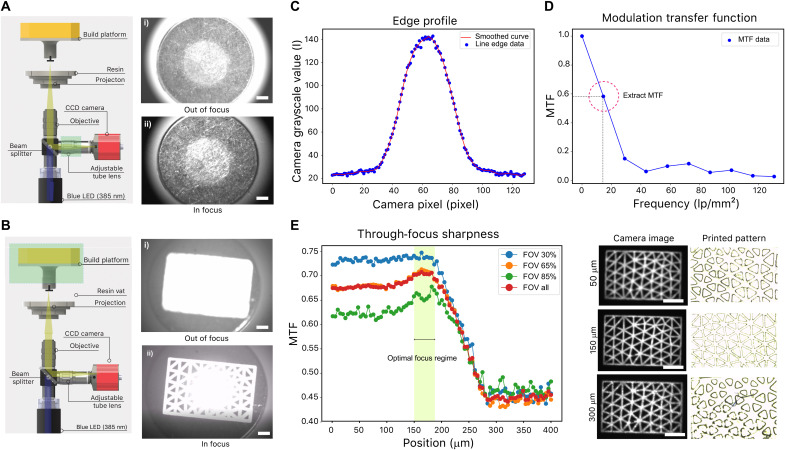
Contrast-based focus algorithm for optimization of the projection focal plane. (**A**) Focus on the build platform with strobe light by finely adjusting the tube lens (highlighted in green). (i) Build platform is out of focus; (ii) build platform is in focus. Scale bars, 2.5 mm. (**B**) Focus on a projected pattern by finely adjusting the vertical position of the build platform (highlighted in green). (i) Projected pattern on the build platform is out of focus; (ii) adjusted build platform brings the projected pattern into focus. Scale bars, 2.5 mm. (**C**) Edge profile of the projected pattern. (**D**) The calculated MTF of the edge profile. (**E**) Through-focus sharpness performance obtained from scanning near a rough estimation of the optimal focal plane of 400 μm. Best focal plane with the highest sharpness performance is found and compared with actual prints. The *z* position with the highest sharpness also has the best resolved 3D print. Scale bars, 1.0 mm.

Because of the shallow depth of focus (14 μm) of high-magnification objectives, we implemented a precise contrast-based focusing mechanism to the 3D printer system to achieve the single-digit-micrometer resolution and optimize the overall projection optics setup. The in-line focusing subsystem consists of a beam splitter, a tube lens, a couple of microscope objective candidates, and a CCD camera (Fig. 2, A and B). This subsystem obtains the through-focus sharpness of the projected pattern, and we verified the performance by comparing the sharpness modulation transfer function (MTF) and the printed pattern side by side. The contrast-based focusing approach demonstrated that we can rapidly and reproducibly achieve our projection optics at the best focal plane for multiple setups containing different microscope objectives (×2 and ×5).

The contrast-based focal plane optimization method contains three separate steps: (i) Coarse tuning I: Roughly tune the adjustable tube lens with strobe light illumination to bring the build platform in focus (Fig. 2A). (ii) Coarse tuning II: Roughly adjust the build platform translational *z* stage to bring the projected pattern in focus (Fig. 2B) and repeat (i) and (ii) until both the build platform surface and the projected image are both in focus. (iii) Fine-tuning: Finely scan through the *z* direction (400 μm) with the translational *z* stage and obtain a through-focus projected image *z* stack (Fig. 2, C to E). More details on the implementation of contrast-based focusing algorithm can be found in Materials and Methods. In the present discussion, we will focus on the analysis scheme that extracts the sharpness information from the projected image.

The MTF calculation that we adopt is similar to the traditional optical transfer function obtained from slanted edge images (*44*). The line edge profile for the projected mesh image is extracted from the center of the strut, and the line spread function (LSF) is calculated with the first-order derivative of the line edge profile. After obtaining the LSF, a fast Fourier transform is applied to the LSF to obtain the MTF in units of line-pair (lp) per square millimeter. We extracted the full field-of-view MTF at a frequency of 12.6 lp/mm^2^ and compared the through-focus MTF to obtain the optimal focal plane. Details on the algorithm can be found in section S1.

Last, we obtained a series of through-focus print results and compared them with our projected image sharpness analysis. The through-focus sharpness results obtained from this analysis corroborate our experimental results (Fig. 2E). From the full scanning distance of 400 μm, we have observed the optimal focus position 150 μm away from the initial scanning position. Because of the shallow depth of focus (14 μm) of high-magnification objectives, it is observed that both the print performance and the sharpness degrade rapidly as we move away from the optimal focal plane. Through this contrast-based *z*-directional focusing method, we can readily identify the optimal focus position for high-resolution 3D printing.

### High-resolution CLIP modeling of optics, momentum, and mass transport

Compared to commercial CLIP 3D printers that have projection optics resolutions of 75 to 160 μm, the newly developed single-digit-micrometer-resolution CLIP-based 3D printer has a target resolution of 1.5 to 3.8 μm. With a 50× improvement of the resolution, it is important to understand the physics of the CLIP printing process and assign the optimal printing parameters to resolve the smallest possible features. To fully understand the high-resolution CLIP printing process, it is critical to study the three key physical models involved: (i) optical model, the estimation of a single-pixel projection width from the projection lens optics; (ii) fluidic model, the uncured resin flow profile during the vertical translation stage movement; and (iii) chemical model, the photopolymerization (curing) including the gradient of oxygen concentration.

**Fig. 3.**
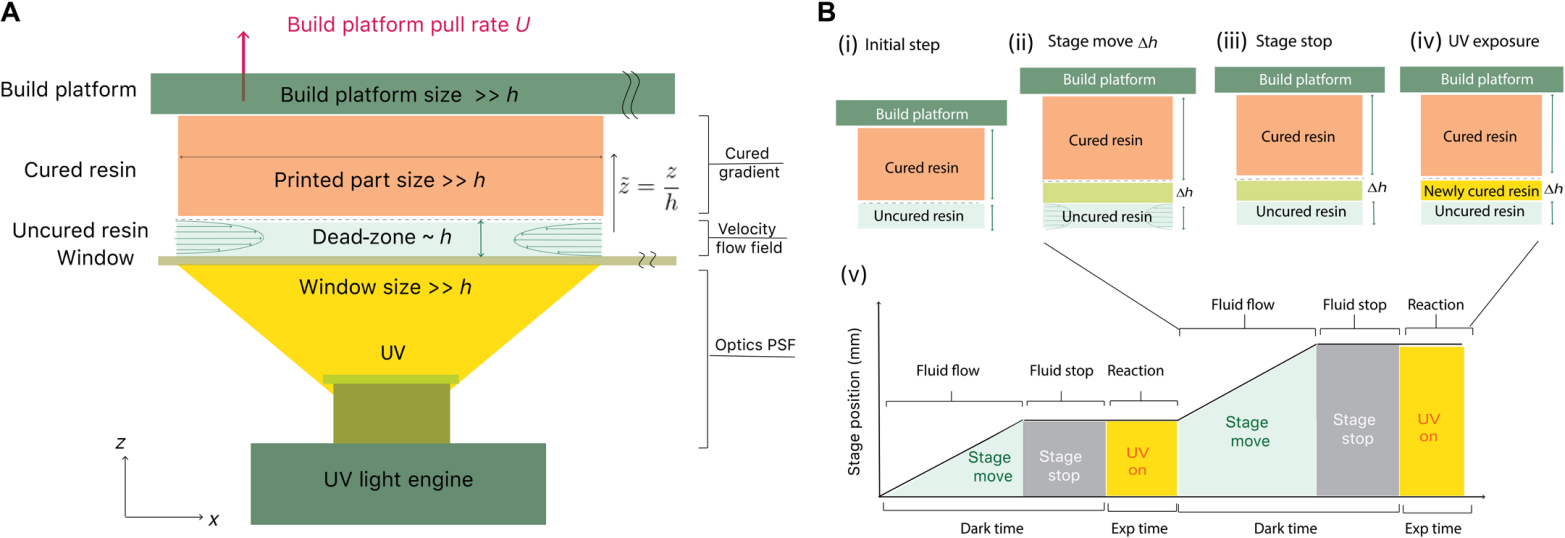
Schematic of CLIP setup and printing process. (**A**) Schematic of a general CLIP printing setup. The setup includes (from bottom to top) a UV light engine that illuminates UV projection at a wavelength of 385 nm, an oxygen-permeable window, a dead-zone (height *h*) where uncured resin flows through, cured resin, and a build platform that travels at a pulling rate *U*. (**B**) Schematic of the stepped printing processes containing the (i) initial step, (ii) stage movement, (iii) stage stoppage, and (iv) UV exposure that are (v) repeated throughout the print process.

The key elements that are involved in a high-resolution CLIP printing process can be found in Fig. 3A. These include (from bottom to top) an ultraviolet (UV) light engine that illuminates UV projection at a wavelength of 385 nm, an oxygen-permeable window, a dead-zone (height *H*) where uncured resin flows through, the cured resin part, and a build platform that travels at a step size ∆*h*, at a pulling rate *U*. We have developed a general model that covers a wide range of CLIP printers with various print resolution capabilities of 30- and 1.5-μm resolution. After developing a numerical model to fully understand the CLIP process, we have adopted the model to an actual 3D printing scenario where the process repeats through three basic steps (stage move, stage stop, and UV exposure; Fig. 3B). Together, the multiphysics simulation tool enables the estimation of many crucial factors required for achieving a successful 3D print, such as the spatial resolution of a 3D printed part, the maximum delamination force experienced for a given projection area, and the dead-zone thickness that is dependent on the printing parameters including dark time, exposure time, and UV intensity (*45*).

#### High-resolution 3D CLIP printing strategy

With the knowledge of the high-resolution 3D CLIP printing process, a general description of the printing strategy is developed as follows. For the first layer of the print, the photocurable resin undergoes a longer initial exposure step that overcomes the gap in between the uncured resin and build platform to allow the resin to successfully bond and attach to the build platform. The remaining layers are repeated through the following processes (Fig. 3B, i). For the *n*th layer of the print, the stage moves upward (*z* direction) by an increment of ∆*h* (layer thickness; typically between 0.5 and 1 μm). This upward stage movement transiently creates a negative pressure within the dead-zone, resulting in resin replenishment from both the left and right ends of the cured part (Fig. 3B, ii) until resin flow into the gap is completed and the uniform pressure is restored. There is a minimum amount of time required for the resin to travel to the center of the build part. Therefore, it is critical to pause after the vertical stage movement to allow for the uncured resin to fully replenish the gap that has thickened by an increment of ∆*h* (Fig. 3B, iii). After the fluid has reached the deepest pixel region (center of the cured resin) and the resin flow is essentially at a quiescent state, we then expose the resin with the UV pattern for the (*n* + 1)th layer, which cures an additional ∆*h* of resin (Fig. 3B, iv). These three steps of printing that involve stage movement, stage stoppage, and UV exposure are repeated until the full 3D print is completed. The overall timeline and the print process are depicted in Fig. 3B (v). The estimation of the required stage traveling time and pausing time are grouped together as the “dark time,” and the duration of this dark time is informed by our model (details can be found in the High-resolution CLIP modeling: Mass transport and print speed section.

#### High-resolution CLIP optics modeling: Projection optics simulation

**Fig. 4.**
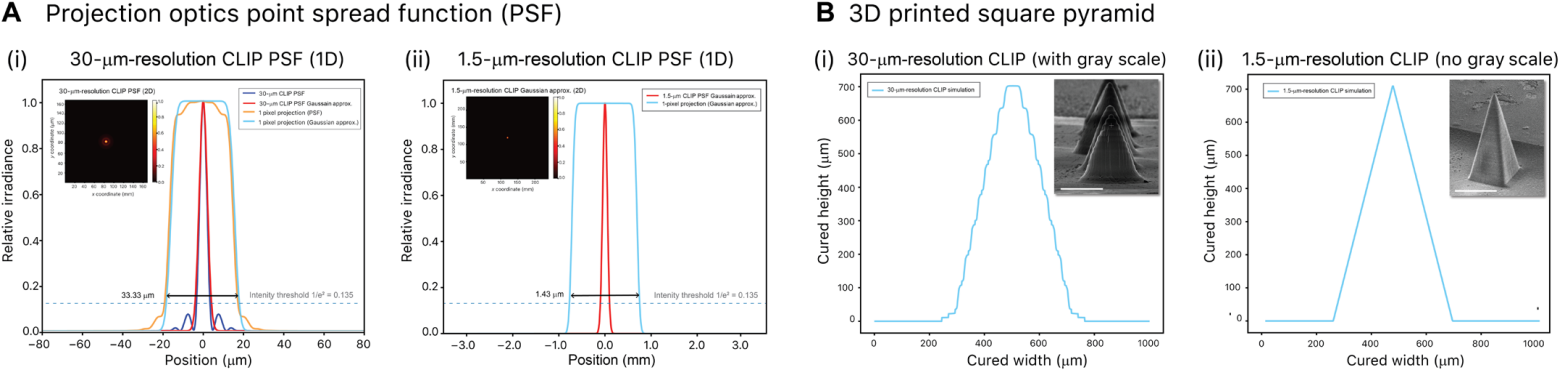
CLIP printing process model includes projection optics, velocity flow field, polymerization gradient, and final 3D printed structure. (**A**) Simulation of PSF from Zemax and Gaussian approximation [for (i) 30-μm-pixel projection lens] and Gaussian approximation[(for (ii) 1.5-μm-pixel projection lens]. Insets are 2D visualizations of the PSF. (**B**) Simulations of a full 3D print that combines optical Gaussian approximation and photopolymerization to predict the overall printing performance of a square pyramid structure (width of 500 μm and height of 1000 μm). Insets are SEM images of an actual 3D printed part for comparison. Scale bars, 250 μm.

Factors that govern the print resolution include the spatial distribution of the projection optics, exposure energy per unit area, and the physical-chemical characteristics of the photopolymer resin and printing parameters (details can be found in section S3). We begin with our focus on the projection optics simulation. The incoherency of the UV-reflected pattern from the DMD of the light engine allows us to model the final energy spread at the projection plane as the superposition of point spread functions (PSFs) of all pixels on the DMD surface via the spatial convolution equation (*46*)

$$\;f(x,y)*\text{PSF}(x,y)=\int_{\tau_{1}=-\infty }^{\infty }\int_{\tau_{2}=-\infty }^{\infty }f(\tau_{1},\tau_{2})∙\text{PSF}(x-\tau_{1},y-\tau_{2})d\tau_{1}{d\tau }_{2}$$ (1)

where *f*(*x*, *y*) is the spatial intensity pattern projected through the DMD. A single pixel *f*(*x*, *y*) is therefore

$$\;f(x,y)=\{\begin{array}{c} \frac{\text{gray scale}}{255}-m\frac{d_{x}}{2}<x<m\frac{d_{x}}{2},-m\frac{d_{y}}{2}<y<\frac{d_{y}}{2}\\ 0\;x<-m\frac{d_{x}}{2},m\frac{d_{x}}{2}<x,y<-m\frac{d_{y}}{2},\;m\frac{d_{y}}{2}<y \end{array}$$ (2)

where *d_x_* and *d_y_* are the lengths of the micromirror along the *x* and *y* axes, *m* is the magnification of the projection optics, and gray scale is the intensity of a single DMD pixel. The spatial convolution equation (Eq. 1) determines the equivalent Gaussian distribution function (ω_0_) in the focal plane of the projection optics. In Fig. 4A, the 2D cross section of the fitted Gaussian curve and the effect of the spatial convolution of a pixel are shown for both the 30-μm-pixel and 1.5-μm-pixel projection optics. Under the assumption that the width of the spot diameter is 1/*e*^2^ of the maximum intensity (*47*), the single-pixel projection width is simulated as 33.3 and 1.42 μm for the 30- and 1.5-μm-pixel projection systems, respectively. Last, the spot diameter at the focal plane can be expressed as the Gaussian distribution, where the UV intensity of an ideal point source on the projection plane at the given position of *x* and *y* is defined by

$$\;I\;(x,y)=\frac{2P}{\pi \omega_{0}^{2}}e^{\left(\frac{-2(x^{2}+y^{2})}{\omega_{0}^{2}}\right)}$$ (3)

where *I* (in J/cm^2^s) is the intensity distribution of the UV light, *P* (in J/s) is the total power of the UV light, and ω_0_ (Gaussian radius) is the half width at the 1/*e*^2^ of Gaussian maximum intensity (*I*_max_). The *x*-*y* plane is the focal plane of the projection optics and is located just above the dead-zone surface in our experimental setup.

#### High-resolution CLIP kinetics modeling: Photopolymerization gradient study

To develop our process model, we first adopt a basic reaction set commonly used to describe free radical polymerization similar to that presented by Dendukuri *et al.* (*48*). In the first step, UV light incident on the sample photolyzes the photoinitiator to produce a pair of radicals. The UV light intensity varies through the sample height according to Beer’s law (*48*)

$$r_{a}=\varphi \varepsilon [\text{PI}]I_{0}\text{exp}(-\varepsilon [\text{PI}]z)$$ (4)

where φ is the quantum yield formation of initiating radicals, ε is the molar extinction coefficient of the photoinitiator at 385 nm, *I*_0_ is the UV intensity at the surface of the window, and [PI] is the concentration of the photoinitiator. Once the oxygen concentration is below a certain threshold, the photoinitiator radicals react with an unreacted monomer to initiate the chain polymerization. In the chain propagation step, the concentration of the growing radicals ($\overset{\cdot }{R},\overset{\cdot }{R}M_{n}$) is lumped into term $\left[\overset{\cdot }{X}\right]$, and their formation propagates through a chain of reactions with other monomer molecules to form a larger radical with rate constant *k*_p_. Then, radicals are consumed and terminated through two separate reactions: (i) chain termination (which is biomolecular in polymer radical) with rate constant *k*_t_ and (ii) oxygen inhibition reaction with rate constant *k*_o_. In this model, only bimolecular termination is considered, while other modes of loss of radical such as trapping of radical species in the resulting polymeric gel are neglected but likely are playing a role to some extent in diminishing the rate of termination relative to propagation (*49*)

$$\;r_{c}=k_{t}{\left[\overset{\cdot }{X}\right]}^{2}+k_{o}[\overset{\cdot }{X}][O_{2}]$$ (5)

By making the quasi-steady approximation (radical concentration remains constant, that is, the rate of initiation equals the rate of termination, *r_a_* = *r_c_*.) to describe the concentration of radicals, we obtain

$$\;[\overset{\cdot }{X}]=\frac{-k_{o}[O_{2}]+\sqrt{{\left(k_{o}[O_{2}]\right)}^{2}+4r_{a}k_{t}}}{2k_{t}}$$ (6)

By constructing the mass transport equations for oxygen and the unconverted oligomer [*M*], we can solve for their spatial-temporal concentration. The oxygen and oligomer transport and consumption inside the system are described by

$$\;\frac{\partial [O_{2}]}{\partial t}=D_{o}\frac{\partial^{2}[O_{2}]}{\partial z^{2}}-k_{o}[O_{2}][\overset{\cdot }{X}]$$ (7)

$$-\frac{\partial [M]}{\partial t}=k_{p}[M][\overset{\cdot }{X}]$$ (8)

No flow is assumed so transport is by diffusion only, and the diffusion of the oligomer is assumed small, so it is neglected.

Nondimensionalizing (Eq. 7) using τ = *tD*_o_/*H*^2^, θ = [O_2_]/[O_2,eqb_], η = *z*/*H*, where *D*_o_ is the diffusivity of oxygen in the oligomer, *H* is the height of the dead-zone thickness, and [O_2,eqb_] is the equilibrium concentration of oxygen at the surface of the oxygen-permeable window, we obtain

$$\frac{\partial \theta }{\partial \tau }=\frac{\partial^{2}\theta }{\partial \eta^{2}}-{\text{Da}}_{1}\theta (-\theta +\sqrt{\theta^{2}+\text{αexp}(-\beta \eta )})$$ (9)

$${\text{Da}}_{1}=\frac{k_{o}^{2}H^{2}[O_{2,\text{eqb}}]}{2k_{t}D_{o}},\alpha =\frac{4\varphi \varepsilon [\text{PI}]I_{0}k_{t}}{k_{o}^{2}{\left[O_{2,\text{eqb}}\right]}^{2}},\beta =\varepsilon [\text{PI}]H$$

In the above, Da_1_ is the dimensionless Damköhler number that quantifies the ratio of oxygen inhibition versus oxygen diffusion into the resin. The boundary conditions and initial condition in our system are

θ(0,τ)=1

$$\frac{d\theta (1,\tau )}{d\eta }=0$$

θ(η,0)=1

where we assume that the initial resin region is oxygenated and the flux of oxygen through the build platform is zero. Since the diffusivity of oxygen in the Teflon AF 2400 window is greater than the diffusivity of oxygen in the resin trimethylolpropane triacrylate (TMPTA), we assume that oxygen can flow freely through the window and oxygen concentration is equal to [O_2,eqb_] at the window surface.

By nondimensionalizing (Eq. 8) with ξ = [*M*]/[*M*_0_], ${\text{Da}}_{2}=\frac{k_{p}k_{O}[O_{2,\text{eqb}}]H^{2}}{2k_{t}D_{o}}$ and setting [*M*_0_] to be the initial monomer concentration, we obtain

$$-\frac{\partial \xi }{\partial t}={\text{Da}}_{2}\xi (-\theta +\sqrt{\theta^{2}+\text{αexp}(-\beta \eta )})$$ (10)

The initial condition is set to be

ξ(η,0)=0

and Da_2_ is a second dimensionless Damköhler number.

**Fig. 5.**
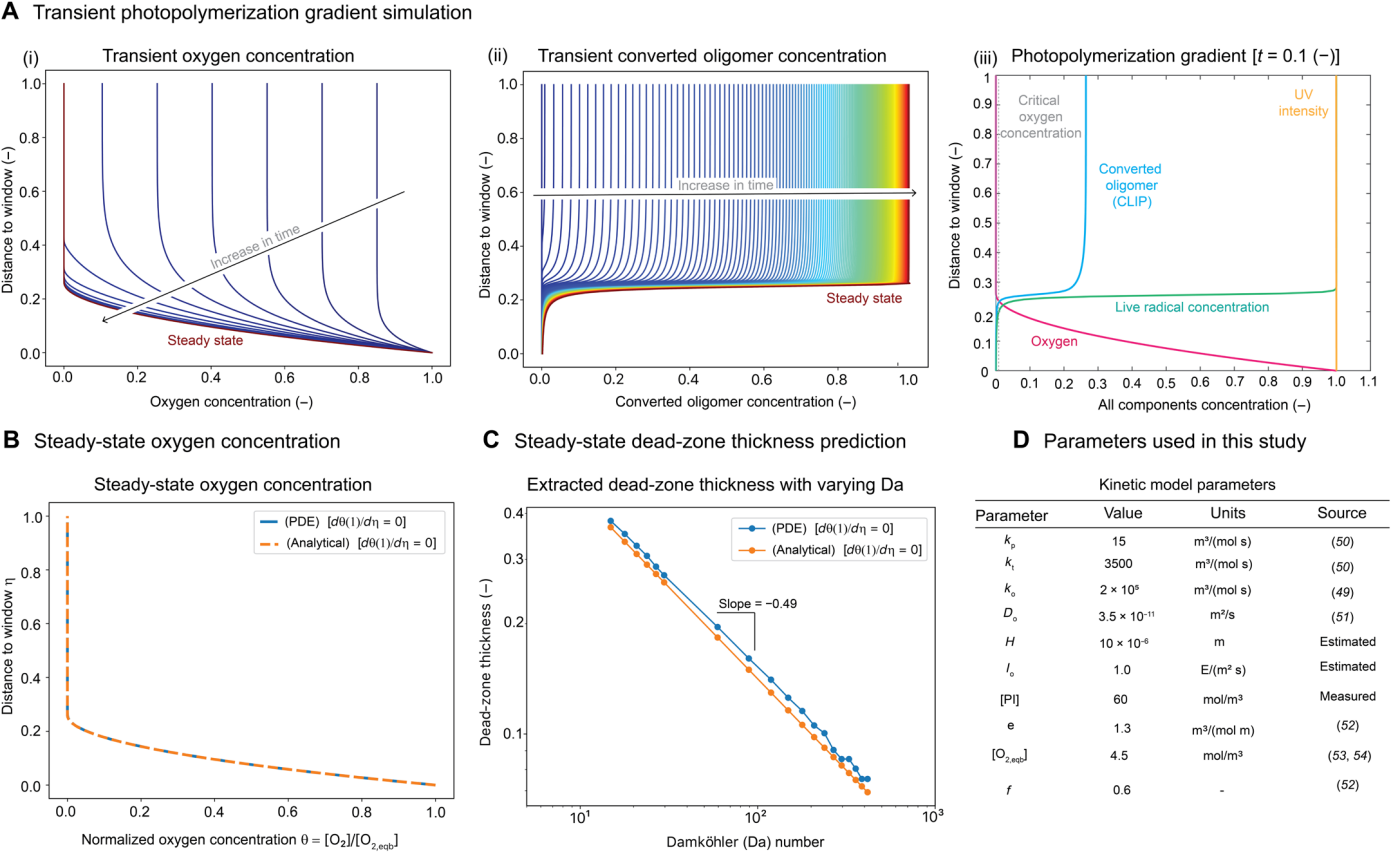
CLIP printing process: Kinetics modeling. (**A**)Transient photopolymerization gradient simulation. (i) Transient oxygen concentration profile in the dead-zone regime. (ii) Transient converted oligomer concentration in the dead-zone regime. (iii) Concentration of all components in the dead-zone regime at *t* = 0.1 (−). (**B**) Steady-state oxygen concentration in the dead-zone regime for both the analytical expression and numerical solution from PDEs. (**C**) Dead-zone thickness at various values of the Damköhler number (Da) from both the analytical expression and the numerical solution of the PDEs. (**D**) Parameters used in this study.

Solving both partial differential equations (PDEs) (Eqs. 9 and 10) simultaneously using the parameters in Fig. 5D, the numerical PDE solution of the transient oxygen concentration and unconverted oligomer concentration can be found in Fig. 5A (i and ii), and a snapshot of all active components within the dead-zone regime at *t* = 0.1 (−) can be found in Fig. 5A (iii). Under the assumption that α = 8.09 × 10^−7^ is small (Fig. 5D) (*50*–*55*), we can obtain the steady-state solution, and an analytical expression for steady-state oxygen concentration is derived and compared with the numerical solution (Fig. 5D). It is found that the numerical steady-state solution matches with the analytical solution, indicating that the steady-state oxygen concentration profile is independent of the boundary conditions at the build platform (details of the full derivation and parameters used can be found in section S3 and Fig. 5). Assuming that the oxygen concentration threshold for the dead-zone (or oxygen inhibition zone) formation is 10^−5^, we can vary the parameter Da_1_ involved in a CLIP printing process and estimate the corresponding dead-zone thickness under different values of Da_1_. It is observed that the dead-zone thickness is approximately 3 to 5 μm from our model prediction, and its thickness scales with the −0.5th power of the appropriate Damköhler number ($\text{Da}={\text{Da}}_{1}\frac{\alpha }{2}$; Fig. 5C)

$$\text{Dead}-\text{zone thickness}\;(h)\propto {\left(\frac{H^{2}\varphi \varepsilon [\text{PI}]I_{0}}{D_{o}[O_{2,\text{eqb}}]}\;\right)}^{-0.5}$$ (11)

The Damköhler number is a measure of the ratio of the rate of oxygen diffusion and the rate of kinetics (photopolymerization and oxygen depletion rate). We provide a justification for this scaling by calculating the steady value of the oxygen concentration (and, hence, the dead-zone thickness) below.

#### Analytical solution for oxygen concentration profile

To provide a deeper understanding of the oxygen concentration profile at steady state, we proceed to solve for an approximate steady-state analytical solution. The dimensionless governing equation for steady-state oxygen concentration is (from Eq. 9)

$$\frac{d^{2}\theta }{d\eta^{2}}={\text{Da}}_{1}\theta [-\theta +\sqrt{\theta^{2}+\text{αexp}(-\beta \eta )}]$$ (12)

but we note that both β and α are small parameters. Because of the fact that β is small, we can approximate Eq. 12 as

$$\frac{d^{2}\theta }{d\eta^{2}}={\text{Da}}_{1}\theta [-\theta +\sqrt{\theta^{2}+\alpha }]$$ (13)

Now, for small values of α, we can expand the term under the radical assuming a Taylor expansion and retain only the leading order term to obtain

$$\frac{d^{2}\theta }{d\eta^{2}}\approx {\text{Da}}_{1}\frac{\alpha }{2}$$ (14)

This is accurate as long as $\theta \gg (\sqrt{\alpha }).$ However, if $\theta \sim O(\sqrt{\alpha })$, the right-hand side of Eq. 13 still represents a positive reaction sink of oxygen. Thus, we conclude that when Eq. 14 is no longer accurate, we enter a regime of approximately zero oxygen concentration. To define that region and connect it to the region of finite oxygen concentration, we apply the boundary conditions

θ(0)=1

$$\frac{d\theta (\tilde{h})}{d\eta }=0$$

$$\theta (\tilde{h})=10^{-5}\sim 0$$

where $\tilde{h}$denotes the end of the region of finite oxygen concentration. Note that the last two conditions are the continuity of oxygen concentration and oxygen flux across the two regions. For values of $\eta >\tilde{h}$[actually values of $\theta \sim O(\sqrt{\alpha })$], we thus assume that the value of θ is essentially zero.

Denoting $\text{Da}={\text{Da}}_{1}\frac{\alpha }{2}$, we can then apply the above boundary conditions to solve for θ in the region of finite oxygen concentration, viz

$$\;\theta =\frac{\text{Da}}{2}\eta^{2}+(-\text{Da}\tilde{h}\;\eta )+1$$ (15)

and we can also solve for the value of ($\tilde{h}$) (which is the steady dead-zone thickness) by using the last condition

$$\;\frac{\text{Da}{\tilde{h}}^{2}}{2}-\text{Da}{\tilde{h}}^{2}+1\sim 0$$ (16)

Solving, we obtain

$$\;\tilde{h}=\sqrt{(\frac{2}{\text{Da}})\;}$$ (17)

This completes the solution, and we demonstrate in Fig. 5C that it is in excellent agreement with the numerical solution for the parameters appropriate to the printing conditions. On the basis of this analysis, an estimation of exposure time for resin to reach steady-state cure height in dead-zone thickness *H* follows the expression

$$t_{\text{cure}}=\frac{H^{2}}{D_{o}}\frac{2\alpha }{{\text{Da}}_{1}}$$ (18)

Overall, this study provides fundamental insights into CLIP printing process, including how the dead-zone thickness depends on the oxygen permeability of the window, the oxygen concentration at the window surface, the applied light intensity, and the photoinitiator concentration.

#### High-resolution CLIP optics and kinetics modeling: 3D print part prediction

We combine our understanding of the photopolymerization model (Eqs. 4 to 17) with the projection optics model (Eqs. 1 to 3) to predict the final 3D printed structure. The photopolymerization model served as a prediction of the cured height, and the 2D projection optics model provided a prediction of the minimum lateral resolution achievable through optical design. To validate the performance of the model, we have designed a square pyramid microstructure and compared the height and surface finish between the 3D printed part from the single-digit-micrometer-resolution CLIP-based 3D printer and the simulated 3D printed object using the simulation model. The simulated results and the corresponding scanning electron microscopy (SEM) images of the 3D printed structures of the 30-μm (Fig. 4B, i) and 1.5-μm (Fig. 4B, ii) CLIP printers are compared to provide a better understanding of how optical resolution can affect the surface finish. The same parameters were used for both the simulation and actual 3D print. The simulation was able to capture the final print height and the surface finish for both high-resolution CLIP printers. The increase in lateral resolution from a 30-μm-resolution to a 1.5-μm-resolution CLIP resulted in a substantially improved surface finish.

#### High-resolution CLIP modeling: Mass transport and print speed

**Fig. 6.**
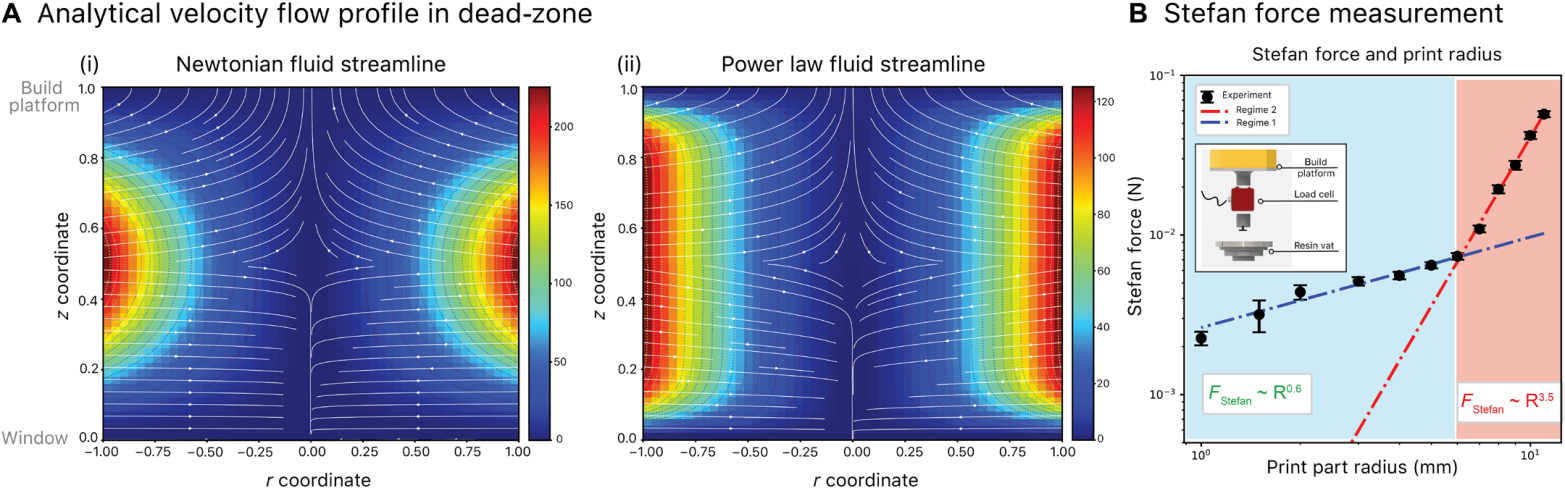
CLIP printing process: Mass transport modeling using lubrication theory. (**A**) (i) Velocity flow profile in the dead-zone regime for Newtonian fluid using the analytical expression derived. (A) (ii) Velocity flow profile in the dead-zone regime for non-Newtonian fluid using the analytical expression derived. Notice that the non-Newtonian fluid is modeled with a power-law fluid with shear-thinning coefficient of *n* = 0.87. (**B**) Direct measurement of Stefan force. Experimental data are obtained through recording the Stefan force during the print process for different 3D print part radius, ranging from 0.5 mm to 2.2 cm. Inset: Load-cell experimental setup for Stefan force measurement.

In this section, we report the development of a predicted flow field during stage movement and an estimation of the force experienced between each print layer. We have modeled the inward flow field of the uncured resin into the gap between the build platform and the window using lubrication theory (*56*). While the “squeeze flow lubrication” for a power-law fluid has been studied previously in the literature (*57*), for completeness, we provide a derivation of the full velocity profile in sections S4 and S5. Note that we have considered both Newtonian and non-Newtonian (power-law) fluids in determining the velocity flow profile and Stefan force in the dead-zone regime. It is worth mentioning that both of our resins show shear-thinning behavior (rheometer data in fig. S3) and are more accurately modeled as power-law fluids. Solving for the analytical velocity flow profile of the Newtonian fluid in the dead-zone regime, we obtain the following equations

$${\tilde{V}}_{r}=-\frac{\tilde{U}}{\varepsilon }\tilde{r}(\tilde{z}(\tilde{h}-\tilde{z}))$$ (19)

$${\tilde{V}}_{z}=-\tilde{U}(3{\tilde{z}}^{2}-2{\tilde{z}}^{3})$$ (20)

where ${\tilde{V}}_{r}$ and ${\tilde{V}}_{z}$ are the dimensionless velocity fields in *r* and *z* directions, $\tilde{U}$ is the dimensionless build platform travel velocity, $\tilde{r}$and $\tilde{z}$ are the dimensionless coordinates in *r* and *z*, $\tilde{h}$ is the dead-zone height, and ε is the ratio of the dead-zone height to the radius of the print part (or build platform; *L*). Solving for the non-Newtonian (power-law fluid) velocity flow profile in the dead-zone regime, we obtain the following for the fields

$${\tilde{V}}_{r}=\{\begin{array}{c} (\frac{1}{\varepsilon })(\frac{\tilde{U}r}{2\alpha })(\frac{1-n}{2-n})[{\left(\tilde{z}-\frac{\tilde{h}}{2}\right)}^{\frac{2-n}{1-n}}-{\left(\frac{\tilde{h}}{2}\right)}^{\frac{2-n}{1-n}}]\;\tilde{z}>\frac{\tilde{h}}{2}\\ (\frac{1}{\varepsilon })(\frac{\tilde{U}r}{2\alpha })(\frac{1-n}{2-n})[{\left(\frac{\tilde{h}}{2}-\tilde{z}\right)}^{\frac{2-n}{1-n}}-{\left(\frac{\tilde{h}}{2}\right)}^{\frac{2-n}{1-n}}]\;\tilde{z}<\frac{\tilde{h}}{2} \end{array}$$ (21)

$${\tilde{V}}_{z}=\{\begin{array}{c} M(\tilde{r})[(\frac{1-n}{3-2n}){\left(\tilde{z}-\frac{\tilde{h}}{2}\right)}^{\frac{2-n}{1-n}}-{\left(\frac{\tilde{h}}{2}\right)}^{\frac{2-n}{1-n}}(\tilde{z}-\frac{\tilde{h}}{2})+\frac{\tilde{U}}{M(\tilde{r})}-(\frac{n-2}{3-2n}){\left(\frac{\tilde{h}}{2}\right)}^{\frac{3-2n}{1-n}}]\;\tilde{z}>\frac{\tilde{h}}{2}\\ M(\tilde{r})[(\frac{1-n}{3-2n}){\left(\tilde{z}-\frac{\tilde{h}}{2}\right)}^{\frac{3-2n}{1-n}}-{\left(\frac{\tilde{h}}{2}\right)}^{\frac{2-n}{1-n}}(\frac{\tilde{h}}{2}-\tilde{z})-(\frac{n-2}{3-2n}){\left(\frac{\tilde{h}}{2}\right)}^{\frac{3-2n}{1-n}}]\;\tilde{z}<\frac{\tilde{h}}{2} \end{array}$$ (22)

where *n* is the shear-thinning power-law exponent obtained from the flow sweep rheology (cf. Supplementary Materials), $M(r)=-(\frac{1-n}{2-n})((\frac{1}{1-n}){\left(\frac{\tilde{U}\tilde{r}}{2\alpha }\right)}^{n}+\frac{\tilde{U}\tilde{r}}{2\alpha })$, $\alpha =2(\frac{1-n}{3-2n}){\left(\frac{\tilde{h}}{2}\right)}^{\frac{3-2n}{1-n}}$, and all the remaining parameters are the same as listed in the derivation for Newtonian fluids section. The velocity profile in high-resolution CLIP for Newtonian fluid based on Eqs. 19 and 20 is shown in Fig. 6A (i) and non-Newtonian power-law fluid based on Eqs. 21 and 22 in Fig. 6A (ii).

As discussed in the “High-resolution 3D CLIP printing strategy” section, we have adopted a stepped printing process (move-stop-expose) to allow for resin replenishment and followed by a quiescent state before curing. It is therefore critical to understand the vertical force experienced during the moving step of each printing process. On the basis of lubrication theory, we calculated the Stefan force involved in each of the print steps. A full derivation of the Stefan force can be found in sections S4 and S5. At present, we focus on the analytical expression and the experimental verification of the Stefan force.

The dimensional Stefan force for both Newtonian and power-law fluids is

$$F_{\text{Stefan}}=\{\begin{array}{cc} -\frac{3\pi \mu U}{2h^{3}}R^{4}\;\text{Newtonian} & \left(23\right)\\ -\frac{\mu_{0}}{4-n}(\frac{U}{2\alpha })R^{4-n}\;\text{non}-\text{Newtonian} & \left(24\right) \end{array}$$

where μ is the viscosity for the Newtonian fluid and μ_0_ is the zero-shear viscosity coefficient for the non-Newtonian fluid (cf. Eq. 16 in section S5). We verify the applicability of lubrication theory on the high-resolution 3D CLIP printer system through directly measuring the force experienced in Fig. 6B. The power-law dependency of the Stefan force with print part radius is observed to be 3.5 when the print part radius is greater than 1.2 cm. When the print part radius drops below 1 cm, the power-law dependencies of 4 − *n* predicted by the lubrication theory cease to hold, and we observe a power scaling of <1 (data analysis scheme can be found in Materials and Methods). This indicates the inapplicability of the simple lubrication theory when print part radius is below 1 cm, although this is still many times the dead-zone thickness (estimation of dead-zone thickness is approximately 3 to 5 μm in the “High-resolution CLIP kinetics modeling: Photopolymerization gradient study” section). Further study in this case as to why the lubrication theory breaks down is necessary and will be the subject of future work.

It has been noticed that if the interlayer time is insufficient for the resin reflow, several print artifacts are expected, such as shorter print height, deformation of print pattern due to liquid flow during incomplete photopolymerization, and surface roughness. For Newtonian fluid, the critical time scale for resin to replenish the vacuum region at the deepest pixel location follows $\frac{h^{2}}{\nu }$and is nearly instantaneous at ~10^−6^ ms, where *h* is the dead-zone thickness and ν is the kinematic viscosity. For non-Newtonian resin, however, the critical time scale is determined by the stress-relaxation time that is related to the %strain that the resin experienced, the evolution of material properties during photopolymerization (*58*), and print diameter. Characterizations on resin stress-relaxation time before (fig. S3) and during printing processes (fig. S4) can be found in the Supplementary Materials. It is observed that to print a structure of 4 to 5 mm in diameter, the relaxation time measured by force sensor measurements is found to be around ~300 to 400 ms, and insufficient stress-relaxation time (<200 ms) for resin relaxation and reflow will lead to a decrease in print resolution (fig. S5).

### High-resolution CLIP print demonstration

**Fig. 7.**
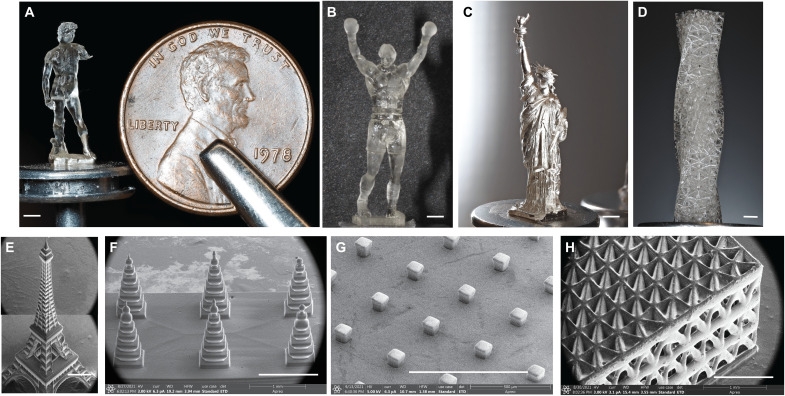
Demonstration prints from the single-digit-micrometer-resolution CLIP-based 3D printer. (**A**) David by Michelangelo (1.2 cm in height) (Florence, Italy). (**B**) Rocky Statue (2 cm in height) (Philadelphia, PA, USA). (**C**) Statue of Liberty (1.5 cm) (New York, NY, USA). (**D**) Lattice twisted bar (1.25 cm in height). (**E**) Eiffel Tower (Paris, France). (**F**) Terraced microneedle. (**G**) Square block array. (**H**) Lattice block. Scale bars, 1 mm.

To demonstrate the capability of our single-digit-micrometer-resolution CLIP-based 3D printer to print intricate, miniature 3D models with a fast speed and high surface smoothness, we explored multiple computer-aided-design (CAD) designs and printed them with various materials. We have explored making 3D structures having a minimum feature size of micrometer length scale (Fig. 7, A to C), a twisted lattice structure 1.25 cm in height with strut thickness of 100 μm (Fig. 7D), and a micro–Eiffel Tower structure 3 mm in height with minimum strut thickness of 50 μm (Fig. 7C). We have also explored structures with engineering and biomedical utility, such as a terraced microneedle design (Fig. 7F) and a square block array (Fig. 7G). All parts in Fig. 7 (A to G) were printed using a resin formulation of TMPTA + 0.3 weight % (wt %) phenol, 2-(5-chloro-2*H*-benzotriazol-2-yl)-6-(1,1-dimethylethyl)-4-methyl (BLS1326) + 2.5 wt % diphenyl(2,4,6-trimethylbenzoyl) phosphine oxide (TPO) and have a viscosity of 0.2 Pa∙s. We have also printed a lattice block with a viscous elastomeric material (EPU-40), with a viscosity of 20 Pa∙s (Fig. 7H). As discussed in the “High-resolution CLIP modeling: Mass transport and print speed” section, it is important to note that the corresponding printing parameters, specifically the interlayer time, are quite different for materials with different viscoelastic properties. The total printing time depends on the height (in *z*) of the design and the viscoelastic properties of the material selected. For example, parts that were printed with a low-viscosity resin, such as microneedle designs or micro square blocks (Fig. 7, F and G), took 3 min to complete. Moreover, the tallest print (2 cm) of a lattice twisted bar design printed with a low-viscosity resin (Fig. 7D) took 90 min. In comparison, a short (2-mm) lattice block printed with a viscous elastomeric resin (Fig. 7H) also took 90 min to complete the print. The increased viscosity of EPU 40 resin and stronger non-Newtonian fluid property indicate that it experiences stronger Stefan adhesion force and requires longer interlayer stress-relaxation time during the print process. This experimentally validates the simulation model regarding the central role of the resin viscosity (Eqs. 23 and 24) and the corresponding Stefan force and resin relaxation time in determining the print speed (detailed discussion can be found in the “Print speed and its role in scalability of high-resolution 3D printing technology” section in Discussion).

### Single-digit-micrometer-resolution and high print speed achieved with high-resolution CLIP 3D printing technology

**Fig. 8.**
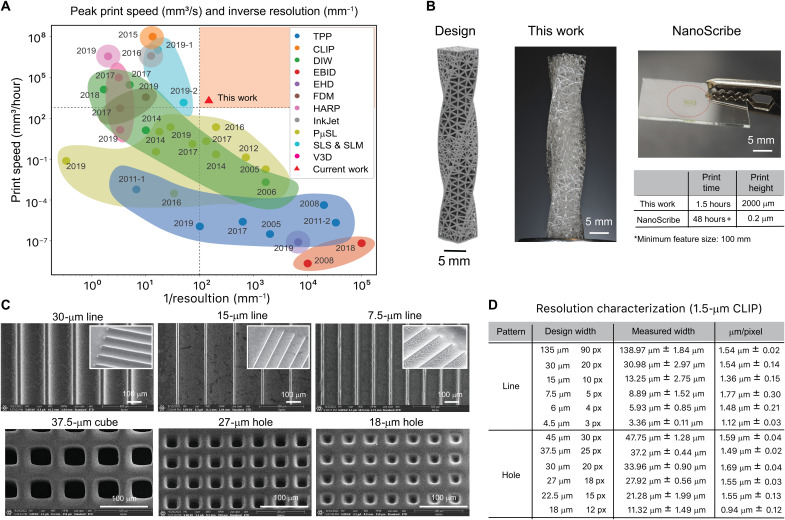
Resolution and print speed characterization. (**A**) Comparison plot of print speed and resolution between high-resolution CLIP and other high-resolution 3D printing technologies. (**B**) Twisted lattice bar. The high-resolution CLIP 3D print and TPP (Nanoscribe, Germany) show that the CLIP technology completed the full print in a much shorter print time compared to the TPP technology. (**C**) Sample images of 1.5-μm-resolution CLIP printer resolution characterization designs for lines of (top row) 30 μm (20 pixels), 15 μm (10 pixels), and 7.5 μm (5 pixels). Insets: Side view of lines resolvability. Bottom row: Holes ranging from 37.5 μm (15 pixels) to 18 μm (12 pixels). (**D**) Summary table of resolution characterization for single-digit-micrometer-resolution CLIP-based 3D printer.

Among current high-resolution 3D printing technologies, it is generally difficult to achieve a high printing resolution while maintaining a high print speed. Nanometer- or micrometer-scale patterning has been achieved with technologies such as electron beam–induced deposition (*47*, *59*), electrohydrodynamic jet printing (*60*), DIW (*25*–*28*), TPP (*15*–*21*), and PμSL (*31*–*41*); however, their print speeds are slow in comparison to IJ (*22*), SLS (SLS and SLM) (*29*, *30*), high-area-rapid-printing (HARP) (*61*), and CLIP printing technologies (*42*, *62*), resulting in lower throughput and scalability. Volumetric 3D printing can achieve faster print speed than TPP, but current geometries and resolution are still limited (*63*–*65*). In comparison to other high-resolution 3D printers, the newly introduced single-digit-micrometer-resolution CLIP-based 3D printer provides a technological platform with high print speed and excellent resolution (Fig. 8A).

To provide a comparison in the practical usage of the newly developed single-digit-micrometer-resolution CLIP-based 3D printer and the commercial TPP printer (Nanoscribe Photonics GT, Nanoscribe, Germany), we have designed and printed a twisted lattice bar structure (2 cm in height with a minimum strut thickness of 100 μm) and compared the total print time and quality. Although TPP is mainly used for nanometer–length scale prints, since there are no other commercially available single-digit-micrometer-resolution 3D printers available, we have thus selected Nanoscribe Photonics GT as a baseline for comparison. While the single-digit-micrometer-resolution CLIP-based 3D printer took 1.5 hours to completely print the 2-cm-height design, TPP took more than 48 hours to print only 200 μm (Fig. 8B). The ratio of the print times is notably around 10^5^ times shorter, highlighting the print speed and ultimate scalability of single-digit-micrometer-resolution CLIP.

2D patterns including lines and holes were designed to characterize and understand the high-resolution printability of our single-digit-micrometer-resolution CLIP-based 3D printer (*66*). Sample SEM images and geometric analyses of the print patterns are presented in Fig. 8C. The full image collection and data analysis scheme can be found in section S2. The designed length scales of tested line patterns range from 135 to 4.5 μm (90 to 3 pixels). It is observed that the print accuracy of the line patterns is optimal at length scales at or above 6 μm (4 pixels), with the dimension precision degrading below this value. As can be observed in the insets of Fig. 8C, the printed lines resolve cleanly for both 30-μm and 15-μm structures, and the border becomes less sharp for 7.5-μm structures, corroborating with the larger SD in print widths. Analysis of micrometers per pixel also showed reductions from our designed projection optics nominal resolution of 1.5 to 1.12 μm per pixel, indicating that the current limit to resolve line patterns is at 6 μm (Fig. 8, C and D). The designed length scale of the tested hole patterns ranges from 45 to 18 μm (30 to 12 pixels). It is observed that the print accuracy of the hole patterns is optimal at 22.5 μm and degrades at a smaller length scale, as shown qualitatively and quantitatively in Fig. 8 (C and D, respectively).

## DISCUSSION

In the present manuscript, we present a single-digit-micrometer-resolution CLIP-based 3D printer that has the capability to generate millimeter-scale 3D prints with single-digit-micrometer resolution in just a few minutes. To achieve the single-digit-micrometer resolution, we have designed and implemented a custom projection lens system that consists of a tube lens and microscope objectives. Moreover, because of the extremely thin depth of field (tens of micrometers) of our high-magnification microscope objectives, we have incorporated a focusing algorithm with an in-line beam splitter and an adjustable tube lens that allows us to visualize the projection pattern with a CCD camera. We have used a digitally designed mesh pattern and have developed a contrast-based algorithm to search for the optimal focal plane position. After scanning through a depth of 400 μm and analyzing the through-focus projected image stacks, we locate the maximum sharpness location and confirm the performance with actual print results. This contrast-based focusing system overcomes the challenge of focusing to the very thin depth of field from high-magnification projection optics and allows us to easily readjust to the optimal focal plane reproducibly.

The resolution performance of the single-digit-micrometer-resolution CLIP-based 3D printer was evaluated through line and hole patterns with the dimensions ranging from 4.5 to 135 μm. While our designed optical resolution is 1.5 μm, the smallest features that were successfully and repeatedly printed were 6-μm lines and 18-μm holes. It is observed that the printer resolution and print performance are strongly dependent on optical resolution, resin formulation, printing strategies, design patterns, and, lastly, cleaning strategies (detailed discussion in the “Achieving 1.5-μm resolution in single-digit-micrometer-resolution CLIP-based 3D printer” section).

Next, we have developed a simulation model that seeks to provide a better understanding of the CLIP printing process and guidance for developing the optimal printing strategies for various materials and designs. The model incorporates an optical simulation of projection optics via a PSF approximated with Gaussian distribution; a prediction of momentum transport and flow field using lubrication theory; and photopolymerization kinetics modeling to predict dead-zone thickness, oxygen concentration gradient, and cured height. The model provides insights to improve the printing process, including adopting a printing strategy of stepped process (stop-move-expose) to allow for an efficient resin reflow, and an estimation of the required interlayer time to eliminate resin convection-induced print artifacts. The model also provides an estimation of parameter values (light intensity and oxygen diffusion coefficient) required to maintain a steady dead-zone for continuous printing. Last, we presented 3D print demonstration of our single-digit-micrometer-resolution CLIP-based 3D printers and the capability to print with viscous elastomeric material.

### Achieving automatic focusing and its role in high-resolution 3D printing technologies

Because of the shallow depth of focus of the highly magnified projection optics system in the single-digit-micrometer-resolution CLIP-based 3D printers and the micrometer-scale features that we are trying to resolve, it is critical to have a robust focusing mechanism to guarantee the print reproducibility and monitor the occurrence of any focus drift. Moreover, it has been observed experimentally that the focal plane of the projection optics at the surface of the dead-zone is strongly dependent on the window material, resin refractive index, and build platform reflectivity. Changing any material in between the path of the projection optics would require a whole new recalibration process. Currently, our method requires several steps of manual focusing, including (i) focusing the CCD camera on the build platform, (ii) focusing the projection pattern onto the build platform, and (iii) obtaining through-focus image stacks to find the optimal *z* location with the best focus performance. While this method has allowed us to achieve single-digit-micrometer resolution, much of the procedure still requires serial steps of manual focusing. It is thus desirable for the single-digit-micrometer-resolution CLIP-based 3D printer to have the capability to autofocus. To achieve autofocus in our current system, a real-time feedback control of a camera imaging system along with a motorized translational *z*-stage actuator for the microscope objective should be designed and implemented. As 3D printing technologies venture into the single-digit-micrometer and nanometer length scales and as autoalignment and multilayer 3D patterning become increasingly common, autofocusing systems have grown into one of the critical components for resolving small print parts.

### Print speed and its role in scalability of high-resolution 3D printing technology

For a long time, despite its many advantages, 3D printing technology was considered a nonscalable manufacturing process because of its limited applications in low-volume production with customized use cases. This was mainly due to the limited resolution and slow print speed of traditional 3D printers. The newly developed high-resolution and high-speed CLIP-based 3D printer introduced here can resolve the main challenges that have been limiting the scalability of AM. With 10^5^ times faster print speeds than the Nanoscribe and 25 to 100 times faster print speeds than DLP and PμSL along with extraordinary resolution performance, the single-digit-micrometer-resolution CLIP-based 3D printer can achieve scalability in many ways that may ultimately elevate the AM industry to a mainstream manufacturing process. These advantages have allowed it to start playing a prominant role especially in biomedical and microelectronic applications, where creating millimeter–length scale 3D objects with micrometer patterning resolution within just a few minutes is highly desirable.

The current dependencies of print speed and resolution can be further explored. It is mentioned in the “High-resolution CLIP modeling of optics, momentum, and mass transport” section that we have adopted a stepped printing process. The print speed is therefore determined by the required interlayer time that includes (i) exposure time and dark time. Dark time consists of (ii) stage travel time and (iii) resin reflow time. On the basis of our kinetics simulation (details can be found in “High-resolution CLIP kinetics modeling: Photopolymerization gradient study” and “Analytical solution for oxygen concentration profile” sections), the exposure time required for our *z* resolution of 0.5 μm is around 1 ms, the stage travel time at stage speed of 1 mm/s and acceleration of 1 mm/s^2^ is measured to be around 50 ms, and the resin stress-relaxation time during the printing process is measured to be in the range of 200 to 1000 ms, depending on the print diameter (section S7). It can be concluded that the major time-consuming step (aka speed-limiting step) is the resin stress-relaxation time in between each layer. While a simple scaling relation between print resolution and print speed is not yet attainable, an experimental measured stress-relaxation time and print diameter relation have been developed (fig. S7B).

It is important to mention that the current print speed can be further enhanced. Methods to reduce the interlayer time include (i) reducing or removing the vacuum pressure experienced at the deepest pixel regime or (ii) increasing the dead-zone thickness. On the basis of Eq. 11, considerations to increase the dead-zone thickness include (iii) increasing the oxygen diffusivity through the oxygen-permeable window or (iv) increasing the oxygen concentration at the window surface. Aside from overcoming the limitation in mass transport within the dead-zone regime, there are other potential methods for developing a complete automatic cycle of a 3D printing process. Streamlining the cleaning, collection, in-line inspection, and storage into a full cycle can help reduce the waiting time and lead to faster turnaround times. The in-line inspection specifically can be bundled with the high-resolution 3D CLIP model development to create a machine learning algorithm that adjusts the printing parameters to achieve the optimal printing accuracy and minimize print defects.

### Print area and its implication for high-resolution 3D printer

One of the trade-offs of UV projection–based vat photopolymerization (e.g., DLP and CLIP) is that as the resolution improves, the overall print area reduces proportionally to the pixel size. For example, for a light engine that has a 2560 × 1600 array of pixels (>4 million pixels), reduction optics method can be applied to modify the pixel size for the user’s selected resolution from a few to hundreds of micrometers so that the corresponding area will be in the range from 5 mm^2^ to 500 cm^2^. There have been several methods introduced to overcome the issue of a limited print area with high-resolution printers. One can implement an *xy*-translational stage that moves the vat by a certain distance to add multiple projections in one layer, similar to the stepper motion introduced in the field of lithography (*67*). Although the overall print area while conserving the high resolution, the overall manufacturing process is slowed down by either the translation stage speed or the postprocessing steps and could introduce additional alignment errors. Recent advances with scanning lens projection methods (*41*) and introduction of lens array (*68*) in PμSL have notably increased the build area and scalability.

### Achieving 1.5-μm resolution in single-digit-micrometer-resolution CLIP-based 3D printer

The difference in theoretical resolution and actual print resolution in *x*, *y*, and *z* is critical to understand our system’s limitation. The theoretical minimum resolvable distance *d* in *x*, *y* resolution in our ×5 objective system, according to Abbe diffraction limit, is $\frac{\lambda }{2\mathrm{NA}}\sim 1.4\;\mu$ (Fig. 4). However, the current achievable minimum *x*, *y* resolution is around 6 μr (Fig. 8). While our current model has only taken into account the light penetration distance in Beer’s law (Eq. 4), we believe that the impact of resin formulation (*45*), reaction-diffusion termination kinetics (*69*), and light scattering of cured parts (*70*) all play a substantial role in *x*, *y* print resolution. The minimum radical diffusion length scale *L* can be used as an estimation of minimum *x*, *y* resolution (*71*). Previous studies have shown that termination reaction coefficient and radical diffusion coefficient are viscosity dependent (*58*, *72*), and we can estimate the diffusion coefficient to slow down on the basis of the degree of conversion and increase in resin viscosity. From preexisting measurements, the estimated radical diffusion coefficient *D* ~10^−9^ m^2^/s (roughly one to two orders of magnitude increase after photocuring) (*73*), and taking the diffusion length scale to be $L=2\sqrt{\mathrm{Dt}}$ and radical lifetime to be *t* ~ 10 to 20 ms, the minimum resolution can be estimated to be around 4.5 to 6.3 μm, which corroborates with our current observations (Fig. 8). Our current modeling has not accounted for reaction-diffusion termination kinetics and light scattering, and future studies on Fourier transform infrared spectroscopy and photorheology will be beneficial to understand the resolution limitation. The *z* resolution in our current system, on the other hand, is only limited by the minimum stage travel distance of 0.1 μm. The prediction of steady-state dead-zone thickness from our kinetics model after the initial exposure is ~3 μ (details of calculation can be found in the “High-resolution CLIP kinetics modeling: Photopolymerization gradient study” section). The dead-zone thickness governs the border of photopolymerization reactions, and the cured height and *z* resolution in each layer are determined by the stage step size ∆*h* after the initial exposure.

### Potential applications with the single-digit-micrometer-resolution CLIP-based 3D printer

Because of the hierarchical nature of the biological systems, microstructures are oftentimes organized and compartmentalized into patterns that are repeated at a length scale several orders of magnitude larger than its minimal subunit. A similar trend is observed in man-made microelectronic systems, in which efficient packaging of multiple layers of micrometer interconnections at the length scale of millimeters is explored. Traditionally, these 3D structures are mainly manufactured by the standard microfabrication methods, such as lithography, etching, and molding (*2*). However, these methods limit design flexibility and production speed. The single-digit-micrometer-resolution CLIP-based 3D printer is capable of manufacturing these microstructures at a similar length scale with more flexibility in design options, allowing additional functions to the microstructures. The capability to create 3D microstructures with single-digit-micrometer features at a speed that is scalable in producing millimeter- and centimeter-size objects is thus one of the key advantages of the single-digit-micrometer-resolution CLIP-based 3D printer and the reason why the newly introduced technology could be very impactful in changing the current landscape at which microarchitectures are mass produced and fabricated. Some examples include microelectronics (*12*–*14*, *25*), microfluidics (*7*, *8*), and microneedles (*4*). Implementing 3D manufacturability to these structures that have had limited designs could add enormous potential to their original functions.

### Postprocessing and its challenges for microstructures

High-resolution 3D CLIP printing has opened the door to unprecedented length scales of microstructures, but cleaning the printed parts has started to show challenges; similar difficulties are involved in traditional lithography when printing structures with high aspect ratios. A known challenge that is related to the capillary-driven collapse of rod structures has been observed during our cleaning process as well (*74*). Fortunately, our 3D printer has the capability to add additional support structures relatively easily to prevent structure collapse, and careful handling of the 3D printed microstructures can oftentimes reduce the issues appreciably. Several improvements can be made to prevent this issue, including increasing the stiffness of the printed material or replacing the isopropyl alcohol (IPA) rinsing solution with a lower–surface tension solvent such as perfluorohexane (*75*). Rinsing procedures that can overcome this failure have been extensively explored in traditional lithographic process, including freeze drying (*76*) and supercritical drying using CO_2_ (*77*). Future directions for rinsing the 3D microstructures can draw inspiration from these already developed methods.

### Simulation model of CLIP-based 3D printing technology and its future direction

Currently several limitations still exist in our multiphysics modeling, and improvements can be further made to the current model.

#### Optic modeling

As discussed in the “Achieving 1.5-μm resolution in single-digit-micrometer-resolution CLIP-based 3D printer” section, it is currently difficult to 3D print patterns at the length scale of the intrinsic pixel size (1.5 μm), and light scattering from cured parts may affect print resolution. Future efforts are ongoing to capture this phenomenon.

#### Kinetics and mass transport modeling

In oxygen transport kinetics modeling, we have not considered the permeability and solubility in resin, and future efforts are ongoing to enrich this assumption. In mass transport modeling, we have assumed that the dead-zone thickness is substantially less than the print length from the lubrication theory. Our result has shown that the lubrication theory is less viable especially for print parts with effective diameter of <1 cm (Fig. 6B), and thus, it is expected that the mass transport model developed here will not be applicable for lattice structures with subcentimeter struts. Solving the full 3D flow profile is therefore the only way to capture the velocity flow field in the dead-zone regime when printed parts are at the similar or smaller length scale of the dead-zone thickness (*56*). While we have considered the non-Newtonian shear-thinning properties of polymeric materials, our current model has neither considered the viscoelastic nature of printing polymeric materials such as EPU-40 (*78*), nor has it considered the evolution of material properties during printing (*58*). Future developments in mass transport model that consider both the intrinsic material properties and the non-Newtonian fluid mechanics arising from photopolymerization will greatly benefit our understanding of printing processes, including a direct estimation of print speed that involves viscoelastic polymeric materials such as hydrogel and elastomers.

Last, there exists great value in the exploration of the projection optics system and fundamental transport phenomena governing the high-resolution 3D CLIP print process. A combination of the digital twin with an in-line 3D scanner or micro-CT inspection system (*79*, *80*) would further allow the development of a machine learning–based model. Using the theoretical model as a baseline to adjust the predicted cured-height landscape and translate to actual dimensions, additional parameters can be captured that cannot be easily simulated by the theoretical model to achieve higher print accuracy.

## MATERIALS AND METHODS

### High-resolution CLIP hardware design

The single-digit-micrometer-resolution CLIP-based 3D printer hardware components can be divided into four components: (i) Projection optics components [light engine (3DLP9000, Digital Light Innovations, TX), tube lens (SM1L10, Thorlabs, NJ; 54-774 Edmund Optics, NJ), and projection lens (5× Mitutoyo Plan Apo Infinity Corrected Long WD Objective for 1.5-μm resolution; 2× Mitutoyo Plan Apo Infinity Corrected Long WD Objective for 3.8-μm resolution; Edmund Optics, NJ)]. (ii) Oxygen-permeable resin vat. A custom-designed 3D printed resin vat with an oxygen-permeable window (Teflon AF2400 film, Random Technology, CA) is the main component for achieving the CLIP technology; it allows the UV to penetrate through for photopolymerization and allows oxygen to permeate through for inhibiting photopolymerization directly above the window within the ~50- to 80-μm thickness of the dead-zone. (iii) Build platform. A high-precision vertical translation stage (GTS70V, Newport, CA) is used to finely adjust the vertical position, and a SEM mount (Ted Pella Inc., Redding, CA) was used as a build platform onto where the printed parts attach. (iv) Real-time projection monitoring subsystem for focusing. The focusing subsystem consists of a beam-splitter cube (CCM1-4ER, Thorlabs, NJ) mounted with UVFS plate beam splitter 30:70 (R:T) (BSS10R, Thorlabs, NJ), a strobe light illumination system (RL3536-WHIIC, Advanced Illumination, Rochester, VT), and a UV camera (CS126MU, Thorlabs, NJ) detector attached to the adjustable tube lens (SM1V10, Thorlabs, NJ) for focusing and monitoring the UV projection.

### High-resolution CLIP software design

Control of the single-digit-micrometer-resolution CLIP-based 3D printer is handled by CLIP3DGUI, a custom software application developed in the Qt framework (Qt Creator, Finland) using C++. CLIP3DGUI controls the operation of the light engine, translation stage, and print process through a set of user-controlled parameters that are optimized for each print.

#### Light engine

One-bit binary image slices (2560 × 1600) generated from the 3D CAD design are imported and then processed with an image encoding pipeline where 1-bit binary images are encoded into 24-bit RGB images, resulting in 24 1-bit images being stored in a single frame. The image encoding results in high image throughput while simultaneously allowing for a low frame rate of streamed images to the light engine, thus avoiding common pitfalls including dropped frames and inconsistent frame rates (*61*). Encoded images are streamed to the light engine through an high-definition multimedia interface (HDMI) cable, and the exposure time, dark time, and light-emitting diode (LED) intensity are all programmatically controlled through lookup tables uploaded to the light engine flash memory.

#### Stage

Translation stage parameters including velocity, acceleration, and jerk must be optimized to allow for swift and precise motion between layers. However, increasing the velocity, acceleration, and jerk parameters also results in larger forces on the part and increased mechanical wear of the translation stage internals.

#### Print process

A series of print process parameters are used to further optimize the print including layer thickness, initial exposure time for adhesion to the build platform, system resync rates, translation stage limits, and starting position.

#### Print script

Exposure time, dark time, LED intensity, stage velocity, and stage acceleration can all be controlled on a layer-by-layer basis to optimize for the exact feature being printed at that time. This allows for the potential of printing vastly different geometries requiring vastly different parameters within the same print.

### High-resolution CLIP materials preparation and resin formulation

The resins used in the experiments were formulated with TMPTA monomer, TPO photoinitiator, and BLS1326 benzotriazole-type UV light absorber, which were all purchased from Sigma-Aldrich (MO, USA). A variety of resins with different mixing ratios were used: 2.5 wt % TPO photoinitiator and 0.3 wt % BLS1326 with TMPTA. A cup of mixed solutions was placed in a THINKY ARE-310 centrifugal mixer (THINKY, CA, USA) and centrifuged for 30 min at 2000 rpm while simultaneously rotating the cup in the opposite direction at 2200 rpm. EPU-40 elastomeric resin was purchased from Carbon 3D (CA, USA). IPA (99%) was used as a rinsing solvent for all printed samples and was obtained from Fisher Scientific (MA, USA).

### High-resolution CLIP materials rheological measurements

The rheological measurements were done on a TA Instrument (ARES-G2) Rheometer (TA Instruments, New Castle, DE). Rheology characterization on TMPTA + 2.5 wt % TPO photoinitiator, 0.3 wt % BLS1326, and EPU-40 were measured. A parallel plate with a diameter of 25 mm was used, and approximately 250 μl of solution was used for each experiment. Characterizations include the following: (i) Flow sweep of the TMPTA resin was done at a temperature of 20°C for soak time of 120 s, with shear rate sweeping from 0.01 to 100 1/s. (ii) Flow sweep of the EPU-40 resin is done at a temperature of 20°C for soak time of 120 s, with shear rate sweeping from 0.01 to 1000 1/s. (iii) Stress-relaxation characterization of the TMPTA resin is done at a temperature of 20°C for a soak time of 60 s, with stress-relaxation duration of 100 s under strain% of 500%. (iv) Stress-relaxation characterization of the EPU 40 resin is done at a temperature of 20°C for a soak time of 60 s, with stress-relaxation duration of 100 s under strain% of 500%.

### High-resolution CLIP Stefan force measurements

Measurements of the Stefan forces experienced by the build platform are conducted using a load cell (FUTEK, Irvine, CA) with a maximum force load of 0.45 kg. The load cell is installed securely in between the build platform, and force readout was conducted at a frequency of 0.005 Hz. The Stefan force is extracted at the regime where the force has reached steady state. The force experienced for each print radius is obtained from calculating the force amplitude readout from each step movement and averaged over 100 s.

### High-resolution CLIP data collection on Nanoscribe

Nanoscribe’s TPP technology was used as a reference to compare the print speed of a high-resolution 3D printer. The 3D structure was printed on a dip-in laser lithography (DiLL) substrate on the Indium-tin oxide (ITO) coated side. The ITO side was identified and confirmed using a multimeter, with a resistance readout of 200 ohms. The objective used to pattern the design is the ×25 objective with the adjustable ring placed at the mark Glyc, and the resin used in this work is IP-S. When printing was completed, the printed part was cleaned by dipping in the propylene glycol methyl ether acetate (PGMEA) developer for 20 min, followed by a quick rinse with IPA, and air-dried with a compressed air gun.

### High-resolution contrast-based focal plane optimization

The contrast-based focal plane optimization contains the following steps. We first perform a coarse adjustment to focus the CCD camera on the build platform. The coarse tuning is performed by fixing the build platform at a specific location while rotating the adjustable tube lens. While we manually flash a strobe light onto the build platform, we focus on the build platform (SEM mount) by tuning the tube lens (Fig. 2A). Figure 2A (i and ii) illustrates the difference between in and out of focus.

Next, we project a mesh pattern from the UV light engine with the minimum feature width of 45-pixel (67.5 μm in ×5 magnification objective and 168 μm in ×2 magnification objective) pattern to an empty vat and drive the build platform in the *z* axis until the projected pattern is roughly in focus (Fig. 2B). Last, we perform fine-tuning with a resin material with the photoinitiator excluded and analyze the through-focus image stacks with a sharpness analysis algorithm (Fig. 2C).

### High-resolution CLIP image slices and 3D printing procedure

The 3D CAD designs were either (i) custom designed using SolidWorks or Fusion360 in-house or (ii) acquired from online repositories (GRABCAD and CGTrader). nTopology (nTopology, NY) was used to generate certain lattice designs. Once the designs were generated, Netfabb (Autodesk, CA) was used to slice them into certain layer thickness. All prints in this study were sliced at a layer thickness of 0.5 μm; as Netfabb could not slice at 0.5 μm, each design was scaled by two in the vertical direction and then sliced at a layer thickness of 1 μm. No modification to the slices was applied. The slices were then applied to our custom software for 3D printing.

## Notes

**Correction (29 November 2022):** The authors note that the righthand labels in Fig. 3A appeared incorrectly. Fig. 3 has been replaced in the PDF and HTML (full text).

## Supplementary Materials

### This PDF file includes:

Sections S1 to S8 Figs. S1 to S5
