# Supplementary material for: Single-digit-micrometer-resolution continuous liquid interface production
Source: Sci Adv. 2022 Nov 16;8(46):eabq2846. doi: 10.1126/sciadv.abq2846 (PMC9668307; doi:10.1126/sciadv.abq2846)
Supplement: Supplementary file 1 — Sections S1 to S8 Figs. S1 to S5 [file sciadv.abq2846_sm.pdf]

Science Advances
AAAS

Supplementary Materials for

Single-digit-micrometer-resolution continuous liquid interface production

Kaiwen Hsiao *et al.*

Corresponding author: Joseph M. DeSimone, jmdesimone@stanford.edu

Sci. Adv. **8**, eabq2846 (2022)
DOI: 10.1126/sciadv.abq2846

This PDF file includes:

Sections S1 to S8
Figs. S1 to S5

Supplementary Text

1. Contrast-based focusing algorithm for searching the optimal focal plane

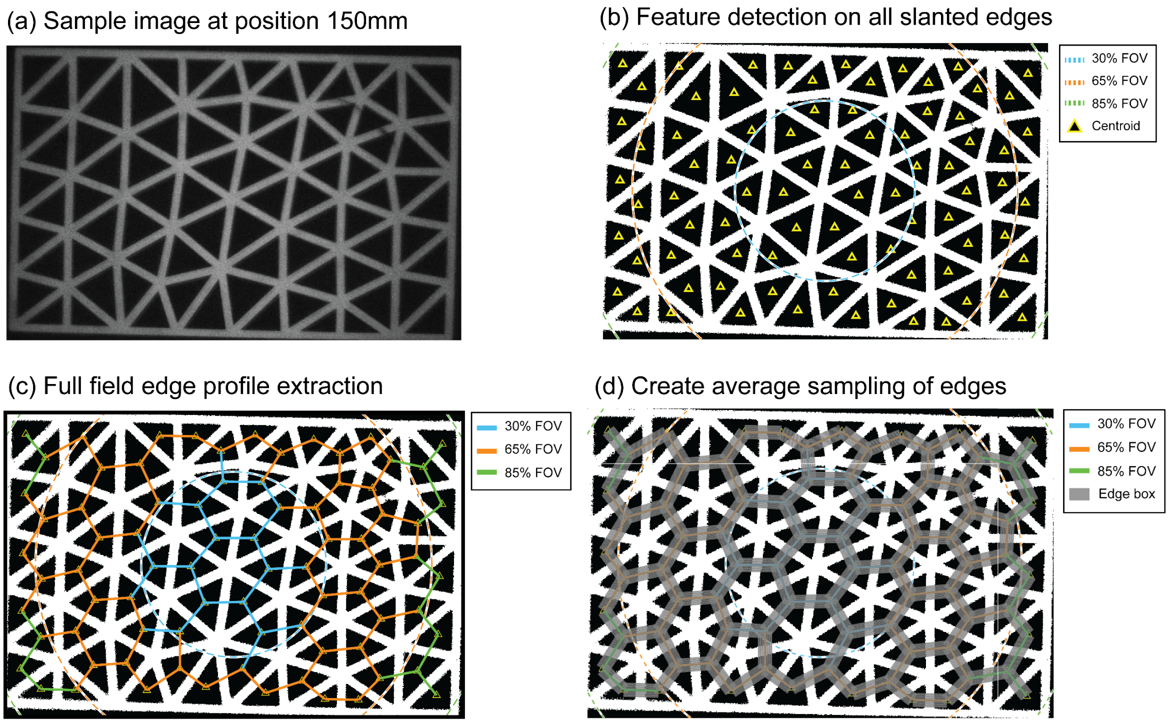

Figure S1. Details on mesh design line edge profiles extraction for through focus contrast-based sharpness analysis. (a) Sample image at a through-focus position 150μm away from the scan starting position. (b) Applied feature detection, extraction, and centroid analysis for full field of view. Field-of-view (FOV) at various distance from the image center are shown. (c) Full FOV edge profile extraction by finding the nearest adjacent neighbors of a given centroid. (d) An average box of width 10 pixels is placed around each sampled edge profile to obtain the average edge profile for each edge.

An image sharpness assessment algorithm is developed to extract the edge profiles within the full field of view (FOV) to obtain the full FOV sharpness analysis. A mesh design is selected for spatial resolution characterization (**Fig. S1**). Feature detection and centroid extraction are applied for finding all triangular elements within the projected image (**Fig. S1(b)**). Distances are then calculated for each centroid, and they are further characterized based on their distance relative to the image center, ranging from 30% FOV, 65% FOV, and 85% FOV. Nearest adjacent neighbor calculation is conducted for each centroid to obtain the line pair for edge profile extraction. The edge profile extraction line segments are color-coded by their respective FOV (**Fig. S1(c)**). Finally, an average box for each line segment with 10 pixels away from the center of the line segment is calculated and plotted (**Fig. S1(d)**). This allows us to obtain the average edge profile for each edge. The obtained edge profile is thus the edge spread function (ESF) required for line spread function (LSF) and modulation transfer function (MTF) for image sharpness through focus.

2. Image analysis scheme for extracting 2D printed feature from SEM images

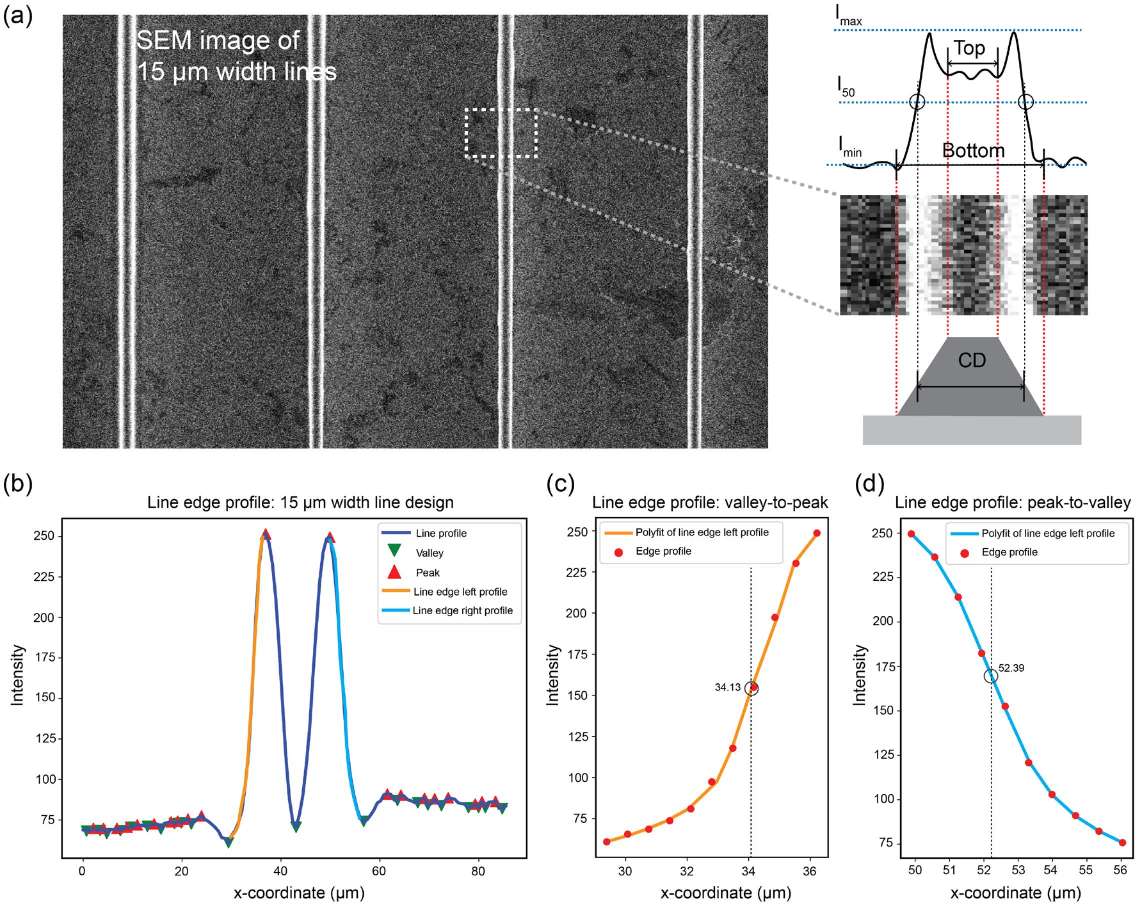

Figure S2. Details on extraction of feature sizes from line edge profiles in SEM images. (a) Critical dimension (CD) extraction using the I_{50} threshold method. (b) Sample line edge profile of a line designed to print at 15µm width. (c) Valley-to-peak intensity profile extraction and polynomial fitting is imposed to obtain the mid-point in the intensity profile. (d) Peak-to-valley intensity profile extraction and polynomial fitting is imposed to obtain the mid-point in the intensity profile

An image analysis scheme is applied to extract the line width from the SEM images. Shown here is a sample line edge profile extracted from a 15µm width line design obtained from SEM. The SEM images are first imported into ImageJ and a single line or hole edge profile is extracted (**Fig. S2**). The edge profile is then subjected to a simple algorithm through peak and valley extraction. The critical dimension (CD) extracted from the SEM is based on the 50% intensity threshold method[66], where the line width reported is based on the x-coordinates extracted at the 50% intensity threshold at the outer edge.

3. Model parameters used in the transport and kinetics model

The final solution of the coupled partial differential equations (PDEs) for both the un-reacted monomer concentration and the oxygen concentration is obtained through MATLAB PDE solver and the parameters used can be found in **Figures. 5(a-d)**. While some parameters are directly obtained through references, there are also values that were estimated or directly measured. We provide a brief discussion of the estimated parameters.

The initial exposure time for resin to cure onto the build platform is roughly around 3s experimentally, depending on the design. Therefore, this gives us a rough estimation of H which should be around 10μm, given that 3s is sufficient for print part to adhere onto the build platform for continuous print to proceed.

Note that while we don't have a direct measurement of the exact light intensity from the 3.5μm or 1.5μm lens, however we did a rough estimation from the intensity that was configured for the 30 μm lens. The intensity I_0 is obtained using the approximation of the known maximum intensity of our light engine for a 30μm printer, which is around 40mW/cm^2. The current value is assumed to linearly scaled by the related Lightcrafter 0-255 control. Nonlinearities in the LED itself as well as temperature fluctuations in the final light intensity have not been considered. To obtain the rough estimation of the light intensity, we took account for the single pixel projected area reduction (30μm: 30; 3.5μm: 3.75; 1.5μm: 1.5) along with f # differences (30μm: 1.3; 3.5μm: 12; 1.5μm: 16). With the listed details, we estimated the initial exposure at UV intensity 1 is 1.1W/m^2 for 3.5μm lens and 4 for 1.5μm lens.

The $[PI]$ concentration is obtained from known photo-initiator concentration 2.5 wt% that is used in our system.

The oxygen concentration at the surface of the window is estimated to be 3 times the concentration of a PDMS surface, due to the fact that a Teflon AF 2400 has 3 times higher permeability to oxygen than PDMS[55]. Further experimental validation of the modeling parameters is crucial for a more accurate prediction. We also note that several key elements of oxygen transport are currently ignored, including the solubility of oxygen in the TMPTA resin as well as the permeability of oxygen through the Teflon AF 2400 window. We use model parameters obtained from known references as a rational framework to understand the CLIP printing process and dead-zone formation.

Thus, we can calculate the critical dimensionless parameters: $Da_1 = 7.34 * 10^7$, $Da_2 = 5.51 * 10^3$, $\alpha = 8.09 * 10^{-7}$, $\beta = 7.8 * 10^{-4}$. We can also obtain the rough estimation of steady state dead-zone thickness as approximately 3 μm given the parameters above.

4. Derivation of lubrication theory applied to CLIP technology – Newtonian fluid

From the CLIP schematic in (**Fig. 4**), the derivation of the lubrication theory after applying the appropriate scaling $\varepsilon \sim \frac{h}{L}$; $\tilde{u}_z \sim \frac{u_z}{U}$; $\tilde{u}_x, \tilde{u}_y \sim \frac{U}{\varepsilon}$; $\tilde{x} = \frac{x}{L}$; $\tilde{y} = \frac{y}{L}$; $\tilde{z} = \frac{z}{h}$ and assuming $\varepsilon \ll 1$, we obtained the simplified governing equations for Newtonian fluid as follows:

Continuity equation:

$$\nabla_s \cdot u_r + \frac{\partial u_z}{\partial z} = 0 \tag{1}$$

Momentum equation:

$$\frac{\partial^2 u_r}{\partial z^2} - \nabla_s p = 0 \tag{2}$$

$$\frac{\partial p}{\partial z} = 0 \tag{3}$$

Where ∇_s is the gradient operator in the $\tilde{x} - \tilde{y}$ plane, The corresponding boundary conditions for the velocity are

$$u_z = \boldsymbol{u}_s = 0, at\ z = 0$$

$$u_z = u_z(h), at\ z = h$$

and $u_z(h)$ is used to describe the velocity of the top plate. From (**Eq. (3)**), pressure is thus only a function of $p^{(0)}(x_s, t)$.

We can then integrate (**Eq. (2)**) and applied boundary conditions

$$\boldsymbol{u}_s = \nabla_s p \left(\frac{z^2}{2} - \frac{zh}{2}\right) \tag{4}$$

To solve for$\boldsymbol{u}_s$, and determine *p* we integrate (**Eq. (4)**) from 0 to h:

$$\int_0^h (\nabla_s \cdot \boldsymbol{u}_s) dz + u_z^{(0)}|_0^h = 0 \tag{5}$$

Applying boundary conditions, we obtain

$$\int_0^h (\nabla_s \cdot \boldsymbol{u}_s) dz = -u_z(h) \tag{6}$$

From (**Eq. (4)**), we know

$$\nabla_s \cdot \boldsymbol{u}_s = \nabla_s^2 p \left(\frac{z^2}{2} - \frac{zh}{2}\right) + \nabla_s p \cdot \left(-\frac{z}{2} \boldsymbol{\nabla}_s h\right) \tag{7}$$

Substituting into (**Eq. (6)**), we obtain

$$u_z(h) = -\int_0^h \nabla_s \cdot \boldsymbol{u}_s dz = \nabla_s^2 p \left(\frac{h^3}{12}\right) + \nabla_s p \cdot \left(\frac{h^2}{4} \nabla_s h\right) \tag{8}$$

Assuming gap thickness h is a constant, we obtain

$$u_z\,(h) = \nabla_s^2 p\left(\frac{h^3}{12}\right) \tag{9}$$

We can further express u_z as

$$u_z = -\int_0^z \nabla_s \cdot \boldsymbol{u}_s d\xi = -\int_0^z \nabla_s^2 p\left(\frac{\xi^2}{2} - \frac{\xi h}{2}\right) d\xi = -\int_0^z \frac{12}{h^3} u_z\,(h)\left(\frac{\xi^2}{2} - \frac{\xi h}{2}\right) d\xi \tag{10}$$

Therefore,

$$u_z = \frac{6}{h^3} u_z\,(h)\left(\frac{z^2 h}{2} - \frac{z^3}{3}\right) \tag{11}$$

If we assume at surface of plate, both velocity and height are maximum and normalized to 1, we can thus set $u_z\,(h) = 1$ and $h = 1$, we then obtain the velocity profile in both x and z direction in the dead-zone regime as follows:

$$u_z = (3z^2 - 2z^3) \tag{12}$$

$$u_r = \frac{1}{\varepsilon} z(z-1) \tag{13}$$

We can solve for the pressure field within the dead-zone regime if we first assume the part footprint is instantaneously a cylinder and L is the radius of the cylinder. Moreover, we assume $p = 0, \tilde{r} = 1$. Integrating (**Eq. (9)**) then gives:

$$P = \frac{12}{h^3}\left\{\frac{r^2 - 1}{4}\right\} \tag{14}$$

We can then integrate the pressure within the circular build area to obtain the Stefan force:

$$F_{stefan}^{Newtonian} = \int_0^R \frac{12}{h^3}\left\{\frac{r^2 - 1}{4}\right\} 2\pi r dr = -\frac{3\pi\mu U}{2h^3} R^4 \tag{15}$$

5. Derivation of lubrication theory applied to CLIP technology – non-Newtonian fluid

For a non-Newtonian power-law fluid we assume. Note that for Newtonian fluid, n = 0.

$$\tau_{rz} = \mu_0 \left(\left| \frac{\partial u_r}{\partial z} \right|^{-n} \right) \left(\frac{\partial u_r}{\partial z} \right) \tag{16}$$

Continuity equation:

$$\nabla_s \cdot u_r + \frac{\partial u_z}{\partial z} = 0 \tag{17}$$

Momentum equation (assuming cylindrical footprint):

$$\tau_{rz} = \frac{dP}{dr}\left(z - \frac{h}{2}\right) \tag{18}$$

$$\frac{\partial p}{\partial z} = 0 \tag{19}$$

Using (**Eq. (16)**) and (**Eq. (18)**) we can obtain expression for $u_r \; at \; z > \frac{h}{2}$:

$$\mu_0 \left(\frac{\partial u_r}{\partial z} \right)^{1-n} = \frac{dP}{dr}\left(z - \frac{h}{2}\right) \tag{20}$$

Solving for u_r with boundary condition $u_r(h) = 0$, we obtain:

$$u_r(z) = \left(\frac{dP}{dr}\right)^{\frac{1}{1-n}} \left(\frac{1-n}{2-n}\right) \left[\left(z - \frac{h}{2}\right)^{\frac{2-n}{1-n}} - \left(\frac{h}{2}\right)^{\frac{2-n}{1-n}} \right] \tag{21}$$

By symmetry we can obtain $u_r \; at \; z < \frac{h}{2}$:

:

$$u_r(z) = \left(\frac{dP}{dr}\right)^{\frac{1}{1-n}} \left(\frac{1-n}{2-n}\right) \left[\left(\frac{h}{2} - z\right)^{\frac{2-n}{1-n}} - \left(\frac{h}{2}\right)^{\frac{2-n}{1-n}} \right] \tag{22}$$

Next, we use the continuity equation to solve for $\frac{dP}{dr}$. From the continuity equation (**Eq. (17)**) we obtain:

$$\frac{1}{2}\frac{\partial}{\partial r}(r u_r) + \frac{\partial u_z}{\partial z} = 0 \tag{23}$$

We can integrate (**Eq. (23)**) from 0 to h in z to obtain:

$$\frac{1}{r}\frac{d}{dr} r \left[\int_0^h u_r dz \right] = -U \tag{24}$$

To get expression for $\left[\int_0^h u_r dz\right]$, we integrate u_r from $\frac{h}{2}$ to h and get:

$$\int_{h/2}^{h} u_r dz = \frac{1}{\mu_0} \left(\frac{dP}{dr}\right)^{\frac{1}{1-n}} \left(\frac{1-n}{3-2n}\right) \left(\frac{h}{2}\right)^{\frac{3-2n}{1-n}} \tag{25}$$

By symmetry, we can express $\left[\int_0^h u_r dz\right]$ as:

$$\int_0^h u_r dz = \frac{-2}{\mu_0}\left(\frac{dP}{dr}\right)^{\frac{1}{1-n}}\left(\frac{1-n}{3-2n}\right)\left(\frac{h}{2}\right)^{\frac{3-2n}{1-n}} \tag{26}$$

We set $\alpha(n) = 2\left(\frac{1-n}{3-2n}\right)\left(\frac{h}{2}\right)^{\frac{3-2n}{1-n}}$ and rewrite (**Eq. (24))** as:

$$\alpha(n)\frac{1}{r}\frac{d}{dr}r\left(\frac{dP/dr}{\mu_0}\right)^{\frac{1}{1-n}} = U \tag{27}$$

After integrating (**Eq. (27)**) and applying boundary condition of $P(0) \to 0$ and $P(R) = 0$, we obtain expression for pressure P as:

$$P = \frac{\mu_0}{2-n}\left(\frac{U}{2\alpha}\right)^{1-n}\left[r^{2-n} - R^{2-n}\right] \tag{28}$$

After we obtained the expression for P, we can then rewrite u_r for $z > \frac{h}{2}$ as:

$$u_r = \left(\frac{Ur}{2\alpha}\right)\left(\frac{1-n}{2-n}\right)\left[\left(z-\frac{h}{2}\right)^{\frac{2-n}{1-n}} - \left(\frac{h}{2}\right)^{\frac{2-n}{1-n}}\right] \tag{29}$$

And u_r for $z < \frac{h}{2}$ as:

$$u_r = \left(\frac{Ur}{2\alpha}\right)\left(\frac{1-n}{2-n}\right)\left[\left(\frac{h}{2}-z\right)^{\frac{2-n}{1-n}} - \left(\frac{h}{2}\right)^{\frac{2-n}{1-n}}\right] \tag{30}$$

We can then solve for u_z at $z > \frac{h}{2}$. Using continuity equation, we can express u_z as:

$$u_z = \int_{\frac{h}{2}}^{z} -\frac{1}{r}\left(\frac{\partial(ru_r)}{\partial r}\right)dz = \int_{\frac{h}{2}}^{z} -\frac{1}{r}\left(\frac{\partial}{\partial r}\left(\frac{dP}{dr}\right)^{\frac{1}{1-n}}\left(\frac{1-n}{2-n}\right)r\right)\left[\left(z-\frac{h}{2}\right)^{\frac{2-n}{1-n}} - \left(\frac{h}{2}\right)^{\frac{2-n}{1-n}}\right]dz \tag{31}$$

For simplicity going forward, we set:

$$M(r) = -\frac{1}{r}\left(\frac{\partial}{\partial r}\left(\frac{dP}{dr}\right)^{\frac{1}{1-n}}\left(\frac{1-n}{2-n}\right)r\right) = -\left(\frac{1-n}{2-n}\right)\left[\frac{1}{1-n}\left(\frac{rU}{2\alpha}\right)^{n} + \frac{U}{2\alpha}\right] \tag{32}$$

We can proceed to solve for (**Eq. (23)**) and apply boundary condition $u_z(b) = U$

$$\frac{U}{M(r)} = \left(\frac{1-n}{3-2n}\left(\frac{h}{2}\right)^{\frac{3-2n}{1-n}} - \left(\frac{h}{2}\right)^{\frac{3-2n}{1-n}} + K_1\right) \tag{33}$$

We thus obtain K_1 as:

$$K_1 = \frac{U}{M(r)} - \frac{n-2}{3-2n}\left(\frac{h}{2}\right)^{\frac{3-2n}{1-n}} \tag{34}$$

We can then obtain u_z at $z > \frac{h}{2}$ as:

$$u_z = M(r)\left[\left(\frac{1-n}{3-2n}\right)\left(z-\frac{h}{2}\right)^{\frac{3-2n}{1-n}} - \left(\frac{h}{2}\right)^{\frac{2-n}{1-n}}\left(z-\frac{h}{2}\right) + \frac{U}{M(r)} - \frac{n-2}{3-2n}\left(\frac{h}{2}\right)^{\frac{3-2n}{1-n}}\right] \tag{35}$$

Similarly, we can obtain u_z at $z < \frac{h}{2}$ with boundary condition as $u_z(0) = 0$:

$$u_z = M(r)\left[\left(\frac{1-n}{3-2n}\right)\left(\frac{h}{2}-z\right)^{\frac{3-2n}{1-n}} - \left(\frac{h}{2}\right)^{\frac{2-n}{1-n}}\left(\frac{h}{2}-z\right) - \left(\frac{n-2}{3-2n}\right)\left(\frac{h}{2}\right)^{\frac{3-2n}{1-n}}\right] \tag{36}$$

Finally, we can solve for the Stefan force for a non-Newtonian fluid to be:

$$F_{stefan}^{non-Newtonian} = \int_0^R \frac{\mu_0}{2-n}\left(\frac{U}{2\alpha}\right)^{1-n}\left\{\frac{R^{4-n}}{4-n} - \frac{R^{4-n}}{2}\right\} 2\pi r dr = -\frac{\mu_0 \pi}{4-n}\left(\frac{U}{2\alpha}\right)^{1-n} R^{4-n} \tag{37}$$

6. Rheological characterization of resins utilized in this work

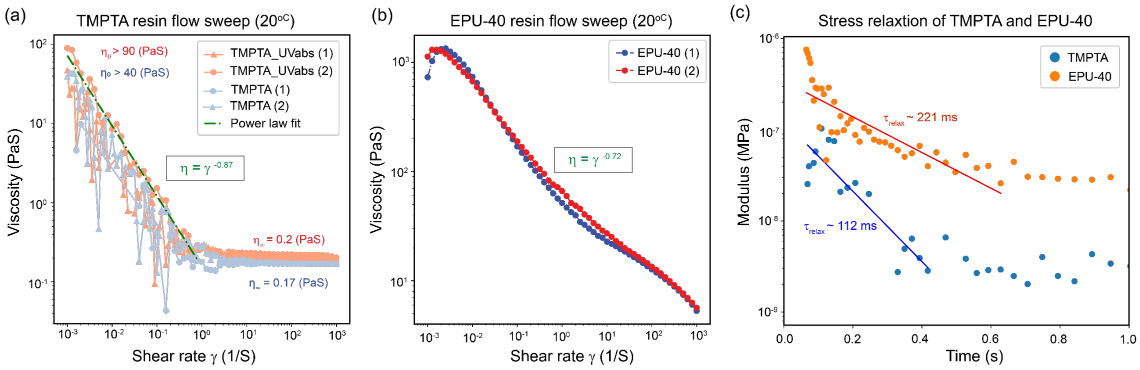

Figure S3. Flow sweep and stress relaxation characterization done on two resins: EPU 40 and TMPTA resin used in this work. (a) Flow sweep of TMPTA resin. (b) Flow sweep of EPU 40 resin. (c) Stress relaxation of uncured TMPTA and EPU 40 resin.

In this work, two resins are used for printing: TMPTA + 0.3wt% BLS1326 + 2.5wt% TPO and EPU 40 (Carbon Inc.). The rheological characterizations including flow sweep of the viscosity as a function of shear rate was performed. A parallel plate device with 25mm diameter was used and approximately 250μL of solution was employed for each experiment.

All rheological properties were characterized using ARES G2 (TA Instruments, New Castle, DE). Flow sweep of the TMPTA resin is done at temperature 20ºC for soak time 120s, with shear rate sweeping from 0.01 to 100(1/s). Flow sweep of the EPU 40 resin is done at temperature 20ºC for soak time 120s, with shear rate sweeping from 0.01 to 1000(1/s). Shear thinning is observed for both TMPTA and EPU 40 resins with different shear thinning coefficients of -0.87 and -0.72, indicating that both resins are non-Newtonian (**Fig. S3 (a), (b)**). Stress relaxation characterization of the TMPTA resin is done at temperature 20ºC for a soak time of 60 s, with stress relaxation duration 100s under strain% 500%. Stress relaxation characterization of the EPU 40 resin is done at temperature 20ºC for a soak time of 60s, with stress relaxation duration 100s under strain% 500%. The longest relaxation time for TMPTA resin is characterized to be 112ms, and the longest relaxation time for EPU 40 resin is characterized to be 221ms (**Fig. S3 (c)**).

7. Resin stress relaxation time and print radius

The transient stress relaxation time required for resin (TMPTA + 0.3wt% BLS1326 + 2.5wt% TPO) with print diameter ranging from 0.4cm to 2.2cm is plotted in **Figure. S5 (a).** The longest relaxation time is extracted by replotting **Figure. S5 (a)** in semi-log plot to extract the average longest relaxation time τ within the transient stress-relaxation process. It is found that the stress-relaxation time increases with increased diameter. Finally, we are aware of the effect of resin shrinkage during curing has a potential impact on the stress-relaxation. However, from Figure. S4 (a) within the 100ms during exposure time, there's no observable relaxation occurring. On-going efforts include using Optical Coherence Tomography (OCT) to better elucidate the effect.

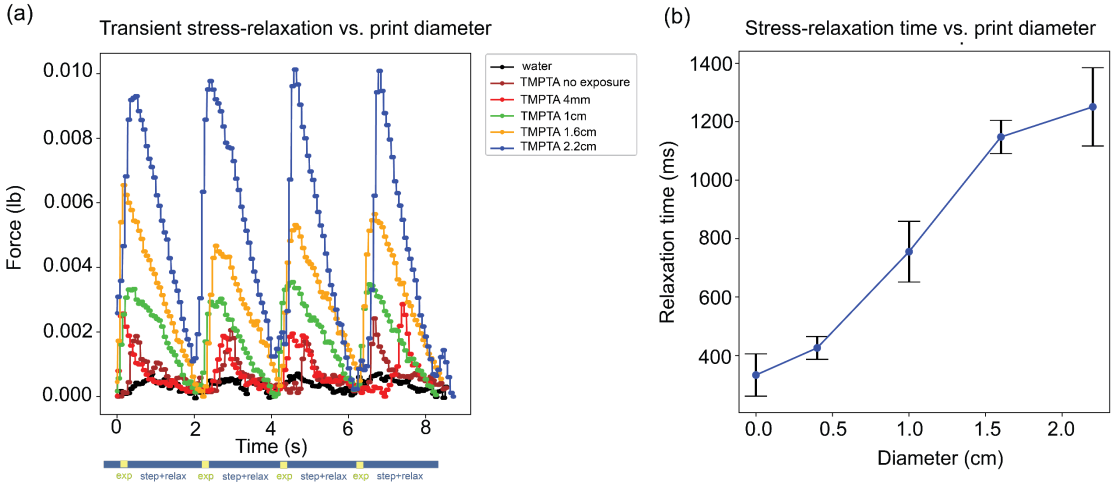

Figure S4. Investigation on the stress-relaxation time for different print diameter. (a) Transient stress-relaxation vs. print diameter for TMPTA resin at different print diameters ranging from dark, 4mm, 1cm, 1.6cm, and 2.2cm. (b) Extracted stress-relaxation time plotted against print diameters.

8. Resin re-flow and print defects under insufficient interlayer time

Based on our prediction on the required time for resin to reflow for the 8.9mm by 5.6mm square area for a resin (TMPTA + 0.3wt% BLS1326 + 2.5wt% TPO) with viscosity approximately 0.2PaS at shear rate of 0.1 (1/s), we have conducted a characterization of the defect versus the interlayer time. The measured interlayer time showed that the defect is gone after approximately at > 200ms (**Fig. S4**).

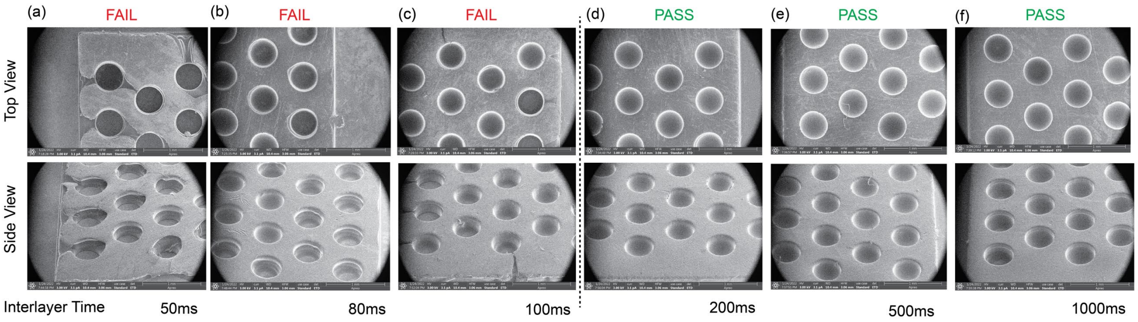

Figure S5. Investigation on the lateral print resolution and the impact of the interlayer time for resin reflow. Interlayer time (a) 50ms, (b) 80ms, (c) 100ms, (d) 200ms, (e) 500ms (f) 1000ms
